# Efferocytosis of viable versus heat-inactivated MSC induces human monocytes to distinct immunosuppressive phenotypes

**DOI:** 10.1186/s13287-023-03443-z

**Published:** 2023-08-17

**Authors:** Michael V. Schrodt, Riley M. Behan-Bush, Jesse N. Liszewski, Madeleine E. Humpal-Pash, Lauren K. Boland, Sabrina M. Scroggins, Donna A. Santillan, James A. Ankrum

**Affiliations:** 1 Roy J. Carver Department of Biomedical Engineering, University of Iowa, Iowa City, IA 52245 USA; 2 Fraternal Order of Eagles Diabetes Research Center, University of Iowa, Iowa City, IA 52245 USA; 3 Department of Obstetrics and Gynecology, Carver College of Medicine, University of Iowa, Iowa City, IA USA; 4 Center for Immunology and Immune Based Diseases, Roy J. and Lucille A. Carver College of Medicine, University of Iowa, Iowa City, IA USA; 5 Department of Biomedical Sciences, Center for Immunology, Center for Clinical and Translational Science, University of Minnesota School of Medicine, Duluth, MN USA; 6 103 S. Capitol St., 5621 SC, Iowa City, IA 52242 USA

**Keywords:** Mesenchymal stem cell, Mesenchymal stromal cell, Efferocytosis, Heat-inactivation, Immunomodulatory, Cell therapy, T-cells, Monocytes, Sepsis

## Abstract

**Background:**

Immunomodulation by mesenchymal stromal cells (MSCs) can occur through trophic factor mechanisms, however, intravenously infused MSCs are rapidly cleared from the body yet a potent immunotherapeutic response is still observed. Recent work suggests that monocytes contribute to the clearance of MSCs via efferocytosis, the body’s natural mechanism for clearing dead and dying cells in a non-inflammatory manner. This begs the questions of how variations in MSC quality affect monocyte phenotype and if viable MSCs are even needed to elicit an immunosuppressive response.

**Methods:**

Herein, we sought to dissect MSC’s trophic mechanism from their efferocytic mechanisms and determine if the viability of MSCs prior to efferocytosis influences the resultant phenotype of monocytes. We cultured viable or heat-inactivated human umbilical cord MSCs with human peripheral blood mononuclear cells for 24 h and observed changes in monocyte surface marker expression and secretion profile. To isolate the effect of efferocytosis from MSC trophic factors, we used cell separation techniques to remove non-efferocytosed MSCs before challenging monocytes to suppress T-cells or respond to inflammatory stimuli. For all experiments, viable and heat-inactivated efferocytic-licensing of monocytes were compared to non-efferocytic-licensing control.

**Results:**

We found that monocytes efferocytose viable and heat-inactivated MSCs equally, but only viable MSC-licensed monocytes suppress activated T-cells and suppression occurred even after depletion of residual MSCs. This provides direct evidence that monocytes that efferocytose viable MSCs are immunosuppressive. Further characterization of monocytes after efferocytosis showed that uptake of viable-but not heat inactivated-MSC resulted in monocytes secreting IL-10 and producing kynurenine. When monocytes were challenged with LPS, IL-2, and IFN-γ to simulate sepsis, monocytes that had efferocytosed viable MSC had higher levels of IDO while monocytes that efferocytosed heat inactivated-MSCs produced the lowest levels of TNF-α.

**Conclusion:**

Collectively, these studies show that the quality of MSCs efferocytosed by monocytes polarize monocytes toward distinctive immunosuppressive phenotypes and highlights the need to tailor MSC therapies for specific indications.

**Supplementary Information:**

The online version contains supplementary material available at 10.1186/s13287-023-03443-z.

## Background

Mesenchymal stromal/stem cells’ (MSCs) immunomodulatory capability and their relative ease of expansion from a variety of tissue sources has made them an attractive option for cell therapy. In fact, MSCs are one of the most widely studied cell therapeutics to date with more than 900 recruiting, active, or completed trials over the past 20 years [1]. They have been studied as a treatment for a variety of disorders, including many inflammatory conditions such as graft-versus-host disease (GvHD), Crohn’s, multiple sclerosis, and, most recently, acute lung injury associated with COVID-19 [2–6].

Despite their widespread use, the mechanisms by which MSCs elicit their immunomodulatory capabilities are diverse and which mechanisms are critical for specific therapeutic applications remain to be identified [7–13]. To date, MSCs have been shown in vitro to suppress inflammation via IDO [14, 15], PGE-2 [16–19], TSG-6 [20–22], and other trophic factors. While inhibition studies have shown these trophic factors play an important role in MSC immune modulation in specific disease settings [23–26], many questions remain. Most critically, how is it that MSCs exert a lasting effect on the immune system when they exist in the body for such a short period of time? MSCs persist only transiently after intravenous infusion, quickly becoming trapped in the microvasculature of the lungs [27, 28], and are then cleared; yet, despite this limited persistence, there is widespread evidence that MSCs lead to durable immune suppression in models of EAE [29, 30], asthma [31], GvHD [18, 32], sepsis [33, 34], and others [35–38]. Recently it has been shown that cleared MSCs are associated with circulating monocytes and tissue-resident macrophages [32, 39, 40]. From this observation a new mechanism of action has been hypothesized: efferocytosis. Efferocytosis is the body’s natural mechanism for clearing dead and dying cells without eliciting an inflammatory response [41–44]. The efferocytosis hypothesis states that MSCs immunomodulatory effects in vivo are largely mediated by monocytes that phagocytose apoptotic MSC debris, after which the monocytes take on an immune resolving phenotype, a process we term efferocytic-licensing. Since this hypothesis has arisen there has been evidence to support the feasibility of it, but many questions remain [8, 45–48].

De Witte et al. [39] demonstrated that after phagocytosis of MSCs, monocytes polarize toward a mixed anti- and pro-inflammatory phenotype with an emphasis toward anti-inflammatory as indicated by gene and surface marker expression. They also observed an increase in Treg production after monocytes phagocytose MSCs. Other studies have shown T-cell suppression in mixed lymphocyte reactions (MLR) when viable, but not non-viable, MSCs are used in a traditional co-culture setup [33, 49]. In these traditional MLR co-cultures, human peripheral blood mononuclear cells (PBMCs) from one donor are cultured with γ-irradiated HLA-mismatched PBMCs of a separate donor and allowed to proliferate for 5–7 days. However, in these studies, efferocytosis-associated immunosuppression was not isolated but instead occurred in tandem with trophic signaling from live MSCs during the T-cell interactions. Because of potential non-efferocytic effects associated with MLR and in vivo studies, determining whether efferocytosis is a benign side-effect of MSC therapy versus a robust mechanism of action of MSC therapy has been difficult to establish.

In this study, we sought to dissect MSCs trophic signaling mechanism from their efferocytic mechanisms to determine if efferocytic-licensing of human monocytes by MSCs leads to an immunosuppressive phenotype and to further determine if the viability of MSCs prior to efferocytosis influences efferocytic-licensing of monocytes. To accomplish this, we efferocytically-licensed human monocytes with either viable- or HI-MSCs and then analyzed the monocytes through a series of phenotypic and functional assays. To ensure we isolate the effect of efferocytosis from the effects of MSC trophic factors, we used a variety of passive and active cell separation techniques to remove non-efferocytosed MSCs before challenging monocytes to suppress T-cells or respond to inflammatory stimuli. The results of this work have implications for assessing the potency of MSCs, the manufacturing of MSC-based therapies, and highlight the importance of host interactions in the success of MSC-based therapies.

## Methods

### Isolation of human PBMCs

Human peripheral blood mononuclear cells (PBMCs) were isolated from a leukapheresis reduction system cone (LRC) of a blood donor from the DeGowin Blood Center at the University of Iowa Hospitals and Clinics by Ficoll-gradient centrifugation. Briefly, human blood from an LRC was flushed with base RPMI and centrifuged at 600*g* without break for 30 min, the buffy coat was collected, and red blood cells were lysed with 1X RBC lysis buffer (1 part 10X RBC Lysis Buffer (Tonbo Biosciences, San Diego, CA, Cat# TNB-4300-L100) + 9 parts sterile DI H_2_O). Isolated PBMCs were further processed for monocyte isolation or cryopreserved in a freezing medium of 50% RPMI 1640 (Gibco, Carlsbad, CA, Cat# 11875-085), 40% FBS (VWR, Radnor, PA, Cat# 97068-085), and 10% DMSO (Fisher Scientific, Waltham, MA, Cat# D128) at 30 million cells per mL. Prior to use in culture, cryopreserved PBMCs were thawed at 37 °C and allowed to reacclimate for 1 h in RPMI containing 10% FBS, 1% L-glutamine, and 1% penicillin–streptomycin (complete RPMI).

### Isolation of human monocytes

Human peripheral monocytes were isolated from PBMCs using a monocyte negative selection magnetic isolation nanobead kit (BioLegend, San Diego, CA, Cat# 480060). Briefly, PBMCs were isolated as described and resuspended to 1 × 10^8^ cells/mL in sorting buffer (1X PBS at pH 7.2, 0.5% (w/v) BSA, 2 mM EDTA). Fc receptors were blocked using 5 µL Human TruStain FcX blocking solution (BioLegend, San Diego, CA, Cat# 422302, provided in monocyte isolation kit) per 100 µL isolation volume for 10 min at room temperature. A 10 µL aliquot of biotin-conjugated antibody cocktail from the isolation kit was then added per 100 µL of cell suspension to label non-monocytes and the cells were allowed to incubate on ice for 15 min. Next, 10 µL of streptavidin-conjugated magnetic nanobeads provided in the isolation kit were vortexed and added per 100 µL of cell suspension. The cells were allowed to incubate on ice for 15 min, then diluted with sorting buffer and centrifuged at 300*g* for 5 min. The supernatant was removed, and the cells were resuspended with 2.5 mL of sorting buffer, then placed in a MojoSort magnet (BioLegend, San Diego, CA, Cat# 480019) for 5 min. After 5 min, the liquid was carefully collected into a clean centrifuge tube and an additional wash with magnetic separation of the beads was performed. Samples of resuspended cells were compared before and after separation by flow cytometry to confirm purity and yield. Isolated monocytes were cryopreserved at 10 million cells per mL using the same freezing media for PBMC cryopreservation. Prior to use in culture, cryopreserved monocytes were thawed at 37 °C and allowed to reacclimate for 1 h in complete RPMI.

### MSC culture

Primary human umbilical cord MSCs (MSCs) were previously isolated from human umbilical cord donors [50]. MSCs were allowed to expand to 80% confluence in MEM-α supplemented with 15% FBS, 1% L-glutamine, and 1% penicillin–streptomycin (complete MEM-α) then harvested on the day of MSC:PBMC co-culture. Passage 5–7 MSCs resuspended in complete RPMI were used for all experiments.

### Heat-inactivation of MSCs

Heat-inactivation (HI) of MSCs was performed by incubating MSCs at 1 million cells/mL in complete RPMI in a 50 °C water bath for 30 min. Following incubation, the cells were centrifuged at 500*g* for 5 min, then resuspended to 1 million cells per mL with complete RPMI.

### MSC efferocytic-licensing

PBMCs were thawed as described above and resuspended at 1 million cells/mL in complete RPMI. They were plated at 200,000 cells per well in a 96-well polypropylene V-bottom microplate (Corning, Corning, NY, Cat# 3357). For experiments requiring various MSC:PBMC ratios, MSCs were resuspended at 1 million cells/mL in complete RPMI, then serially diluted 1:1 with fresh complete RPMI to obtain stock dilutions of MSCs for plating MSC:PBMC coculture conditions of 1:5, 1:10, 1:20, 1:40, and 1:80 in the 96-well polypropylene V-bottom microplate. Conditions consisted of PBMC alone, PBMC + viable MSC, and PBMC + HI-MSC. For samples that do not contain MSCs, an appropriate volume of media was added to maintain culture volume consistency. The co-cultures were allowed to incubate for 24 h before further use or analysis.

For experiments requiring a fixed 1:5 ratio of MSC:PBMC, viable or HI-MSCs were resuspended at 1 million cells/mL in complete RPMI and added to PBMCs (40,000 MSCs: 200,000 PBMCs) in 96-well polypropylene V-bottom microplates. Samples were allowed to incubate as described above.

For experiments requiring a fixed number of MSC:monocytes, viable or HI-MSCs were resuspended at 1 million cells/mL in complete RPMI and added to isolated monocytes at a 1:1 ratio (40,000 MSC: 40,000 monocytes) in 96-well polypropylene V-bottom microplates. Each condition to be tested was plated to separate plates and samples were allowed to incubate as described above.

### Phagocytosis assay

MSCs were stained with CellBrite Orange Cytoplasmic Membrane Dye (CBO) (Biotium, Fremont, CA, Cat# 30022) prior to culture with PBMCs. Briefly, MSCs were resuspended at 1 million cells/mL in complete RPMI and stained with 5 µL of CBO per 1 million MSCs. The MSCs were allowed to incubate at 4 °C for 20 min, and then washed twice with complete RPMI. Following staining, some MSCs underwent heat-inactivation as described above. Stained MSCs were added to PBMCs as described in “MSC Efferocytic-Licensing” above for conditions requiring various ratios of MSC:PBMC.

Following efferocytic-licensing the samples were centrifuged at 500*g* for 5 min, then resuspended with 50 µL pre-made Fc receptor blocking solution per sample (9 parts Cell Staining Buffer (BioLegend, San Diego, CA, Cat# 420201)+1 part Human TruStain FcX (BioLegend, San Diego, CA, Cat# 422302)) (Table 1). Samples were allowed to incubate at room temperature for 10 min. 50 µL of stain index optimized anti-human CD14-AlexaFluor 488 (BioLegend, San Diego, CA, Cat# 325610) (Table 1) antibody was then added to the samples and they were allowed to stain for 30 min at 4 °C in the dark. Samples were washed twice and then analyzed by flow cytometry to detect CD14 + monocytes that had phagocytosed CBO-stained MSCs.

**Table 1:** Product and staining information for on-target, isotype, and blocking antibodies used for experiments

Antibody target	BioLegend Cat#	RRID#	Fluorophore	Isotype RRID#	Conc. (µg/mL)
CD3	317334	AB_2561452	PE-Cy7	AB_2864288	1.25
CD14	325610	AB_830683	Alexa 488	AB_2890263	3.00
CD16	302067	AB_2876587	PE-Fire640	AB_2937018	1.00
CD86	305419	AB_1575070	PerCP-Cy5.5	AB_2937017	0.25
CD90	328110	AB_893433	PE	AB_326435	2.50
CD163	333614	AB_2562641	PE-Cy7	AB_326448	2.00
CD206	321130	AB_2616867	PE-Dazzle594	AB_2923261	3.00

The antibodies listed are individual items and their associated isotypes. Listed staining concentrations are stain-index optimized for the on-target antibodies and were used for the on-target and associated isotype antibodies. Any antibody products that were used as part of a kit are not listed in this table (see “Isolation of Human Monocytes” and “Depletion of Non-Phagocytosed MSCs” sections)

### T-cell proliferation assay

To track T-cell proliferation, PBMCs were thawed as described before and then stained prior to MSC efferocytic-licensing with CFSE Cell Division Tracker Kit (BioLegend, San Diego, CA, Cat# 423801) using the manufacturer’s protocol with optimized staining concentration for use with a Cytek Northern Lights flow cytometer. Briefly, PBMCs were resuspended at 1 million cells/mL in PBS and stained with 1 µL of 0.25 mM CFSE per 2 million PBMCs for a final concentration of 0.125 mM. The PBMCs were allowed to incubate at 37 °C for 15 min, centrifuged at 500*g* for 8 min, resuspended at 1 million cells per mL with complete RPMI, and allowed to incubate at 37 °C for 30 min. PBMCs were centrifuged once more and resuspended to 1 million cells/mL with complete RPMI. MSCs and PBMCs were plated for efferocytic-licensing as described before for experiments requiring various ratios of MSC:PBMC or for experiments requiring a fixed 1:5 ratio of MSC:PBMC.

Following efferocytic-licensing, each culture condition tested (PBMC only, PBMC + MSC, and PBMC + HI MSC) was separately pooled, counted, and resuspended in complete RPMI to 1 million PBMCs/mL. The cells were either further processed to remove non-phagocytosed MSCs or plated to a 96-well flat-bottom polystyrene microplate. For samples that were plated without further processing, 200,000 PBMCs were added per well in triplicate for each condition.

T-cell activation was performed by adding Human T-cell CD3/CD28 Activator Dynabeads (Gibco, Waltham, MA, Cat# 11132D) to PBMCs using the manufacturer protocol with modifications. Briefly, a solution of diluted Dynabeads was prepared by first washing the beads in PBS followed by resuspension in complete RPMI to a concentration of 6X the final concentration. Each sample needing activation received 40 µL of resuspended Dynabeads to achieve a culture volume of 240 µL and a final concentration of half the manufacturer recommended concentration of Dynabeads per activated sample. Non-activated samples received 40 µL of complete RPMI and served as negative controls.

Samples were allowed to incubate for 4 days after which they were collected, and samples were resuspended to disrupt Dynabeads. The samples were placed on a magnet tube rack for 5 min to pull out the Dynabeads and the cells were transferred to new tubes for centrifugation at 500*g* for 5 min. The cells were resuspended with 50 µL pre-made Fc receptor blocking solution per sample as described in Phagocytosis Assay above. Samples were allowed to incubate at room temperature for 10 min. Following incubation, each sample received 25 µL of pre-made cyanine-dye blocking solution (4 parts Cell Staining Buffer + 1 part True-Stain Monocyte Blocker [BioLegend, San Diego, CA, Cat# 426103) (Table 1)]. The samples were each stained with 25 µL of pre-made stain index optimized anti-human CD3-PE/Cy7 antibody (BioLegend, San Diego, CA, Cat# 317334) (Table 1) for 30 min at 4 °C in the dark. Samples were washed twice and resuspended in Cell Staining Buffer prior to analysis by flow cytometry. T cells were gated based on positive CFSE and CD3 signal.

### Depletion of non-phagocytosed MSCs

To remove non-phagocytosed MSCs after efferocytic-licensing, but prior to T-cell activation, MSCs were labeled with anti-human CD90-PE antibody (BioLegend, San Diego, CA, Cat# 328110) (Table 1) then depleted using BioLegend’s MojoSort Human anti-PE Nanobead selection kit (BioLegend, San Diego, CA, Cat# 480092). For consistency, every culture condition (PBMC only, PBMC + MSC, PBMC + HI-MSC) underwent this MSC selection process. In brief, cells were centrifuged at 500*g* for 5 min and resuspended at 10 million cells/100 µL in nanobead selection buffer (1X PBS, pH 7.2; 0.5% (w/v) BSA; 2 mM EDTA). Human TruStain FcX from the selection kit was used to block Fc receptors by adding 5 µL of human TruStain FcX per 100 µL cell suspension at room temperature for 10 min. Anti-human CD90-PE was added to the cell suspension and the cells were stained on ice for 15 min. Cells were washed with nanobead selection buffer and centrifuged at 300*g* for 5 min. Cells were resuspended in 100 µL buffer, a sample was obtained to assess baseline MSC:PBMC ratio by flow cytometry, then 10 µL/10 million cells of human anti-PE nanobeads from the selection kit was added to the remaining cell suspension, and the cells were incubated on ice for 15 min. The cells were washed with buffer and centrifuged at 300*g* for 5 min. The cell suspension was resuspended with 2.5 mL of buffer and placed in a MojoSort magnet (BioLegend, San Diego, CA, Cat# 480019) for 5 min. Unlabeled cells were collected and a sample was obtained to assess effectiveness of MSC removal by comparing to the baseline MSC:PBMC ratio (Fig. 3b, Additional file 1: Figure S3B). The cells were counted, resuspended to 1 million cells/mL in complete RPMI, 200,000 cells were plated per well of a 96-well polystyrene flat-bottom microplate, then T-cells were activated as mentioned above.

### Monocyte phenotyping

To determine monocyte phenotype, triplicate samples of each culture condition tested were plated as described above for experiments using a fixed 1:5 MSC:PBMC ratio. Each triplicate sample of a given condition consisted of 5 replicate wells that were pooled together after efferocytic-licensing. Samples were centrifuged, and Fc receptors and non-specific cyanine dye were blocked using the same protocol used prior to antibody staining in the T-Cell Proliferation Assay method above. Using stain index optimized antibody concentrations, the samples then received 25 µL of pre-mixed phenotype staining solution (CD14-AlexaFluor 488 (BioLegend, San Diego, CA, Cat# 325610), CD16-PE/Fire 640 (BioLegend, San Diego, CA, Cat# 302068), CD86-PerCP/Cy5.5 (BioLegend, San Diego, CA, Cat# 305419), CD163-PE/Cy7 (BioLegend, San Diego, CA, Cat#333614), and CD206-PE/Dazzle594 (BioLegend, San Diego, CA, Cat# 321130)) (Table 1) for 30 min at 4 °C in the dark. Samples were washed twice and resuspended in Cell Staining Buffer prior to analysis by flow cytometry. Cells were gated based on singlet discrimination and CD14 for all monocytes, CD16 was used with CD14 to gate for monocyte subsets (CM, IM, and NCM), and then each subset was gated on CD86, CD163, and CD206 monocyte polarization markers (Additional file 1: Figure S4).

### *Monocyte activation (*in vitro* sepsis model)*

Monocytes were activated by supplementing complete culture media with recombinant IL-2, IFN-γ, LPS, and L-tryptophan. Briefly, following efferocytic-licensing, all wells from the microplates prepared for MSC:monocyte cocultures were pooled according to condition and resuspended to 1 million cells per mL. Each condition of cells was resuspended to 1 million cells/mL in either monocyte activation media or control media. The activation media was prepared by supplementing complete RPMI with recombinant human IL-2 (PeproTech, Cranbury, NJ, Cat# 200-02), recombinant human IFN-γ (PeproTech, Cranbury, NJ, Cat# 300-02), LPS O55:B5 (Sigma-Aldrich, St. Louis, MO, Cat# L6529), and L-tryptophan (Sigma-Aldrich, St. Louis, MO, Cat# T0254) to final concentrations of 5 ng/mL, 10 ng/mL, 10 ng/mL, and 200 µM, respectively. Control media was prepared by supplementing complete RPMI with L-tryptophan to a final concentration of 200 µM. Samples were plated in a 96-well flat-bottom polystyrene microplate with 200,000 monocytes only, 200,000 viable or HI-MSC efferocytically-licensed monocytes, or 40,000 MSCs (viable or HI) per well. Each condition was plated in triplicate and allowed to incubate at 37 °C for 2 days. Media was collected from each sample after 2 days for further analysis.

### ELISA

TNF-α, IFN-γ, and IL-10 soluble protein levels were analyzed using ELISA kits (BioLegend, San Diego, CA, TNF-α: Cat# 430204, IFN-γ: Cat# 430104, IL-10: Cat# 430604). Briefly, 100 µL of diluted capture antibody was added to a 96-well uncoated ELISA plate (BioLegend, San Diego, CA, Cat# 423501) for incubation at 4 °C overnight. The next day, the capture antibody was removed, and the plate was washed 4 times with 300 µL/well of 1X wash buffer (19 parts DI H_2_O + 1 part 20X ELISA Wash Buffer (BioLegend, San Diego, CA, Cat# 421601)). All subsequent wash steps were performed the same. Non-specific binding was blocked by incubating the plate with 200 µL of Assay Diluent from the ELISA kit per well for 1 h with orbital shaking at room temperature. After blocking, the plate was washed again, then 100 µL of standards and samples were added per well. Samples were diluted in Assay Diluent if necessary. The plate was placed back on the orbital shaker for a 2 h incubation at room temperature. Next, the wells were washed again and 100 µL of diluted Detection Antibody from the kit was added to each well, then the plate was returned to the shaker for a 1 h incubation at room temperature. The plate was washed again, followed by addition of 100 µL of diluted Avidin-HRP solution per well. The samples were placed back on the shaker for a 30-min incubation at room temperature. The plate was washed once more, this time 5 times with each wash iteration allowed to soak for 1 min. 100 µL of TMB substrate was then added to each well and the plate was allowed to incubate for 15–45 min in the dark. To stop the reaction 100 µL of 1N H_2_SO_4_ was added to each well. Absorbance was read at 450 nm on a plate reader. Non-specific background subtraction was performed by additionally reading the plate at 570 nm.

### Kynurenine assay

IDO activity was assessed using a kynurenine colorimetric assay. Briefly, L-kynurenine stock was prepared by dissolving L-kynurenine (Sigma-Aldrich, St. Louis, MO, Cat# K8625) in complete culture media at 5000 µM. A 500 µM top standard was prepared by diluting one part stock in nine parts complete culture media, then six 1:1 serial dilutions were performed. 30%(w/v) trichloroacetic acid (TCA) (Sigma-Aldrich, St. Louis, MO, Cat# T9159) was used to precipitate proteins from the standards and sample media at a 1:1 dilution of TCA to media in a 96-well V-bottom microplate. The plate was then heated at 52 °C for 30 min to convert N-formylkynurenine to kynurenine followed by centrifugation at 1200*g* for 15 min to remove precipitated proteins. The supernatant from each sample was split equally into replicate wells of a new 96-well flat-bottom microplate and each well received 0.8%(w/v) Ehrlich’s reagent (4-(Dimethylamino)benzaldehyde in acetic acid) (Sigma-Aldrich, St. Louis, MO, Cat# 156477) at a volume equal to the media volume. Samples were allowed to incubate at room temperature for 10 min and then read on a plate reader at 492 nm. Sample concentrations were interpolated from the L-kynurenine standard curve.

### Flow cytometry controls and antibody information

Instrument and gating controls were prepared using stained MSCs and/or PBMCs under the conditions tested in experiments. In brief, unstained and single-color controls were used to establish instrument voltage settings, isotype controls were used to determine non-specific staining of on-target antibodies, and experiments with more than two fluorophores or dyes used fluorescence-minus-one controls with stain-index optimized on-target antibodies and dyes for establishing gates. If experiments used one or two fluorophores or dyes, then antibody isotype controls or no stain controls were used to establish gates for antibody staining or dye-based staining, respectively. Antigen-fluorophore pairs were deliberately chosen to allow for maximal spectral emission separation within experimental parameters and fluorophores were paired with antigens according to fluorophore brightness and expected antigen abundance.

SpectroFlo Cytometer QC Beads (Cytek, Fremont, CA, Cat# N7-97355-0A) were used for quality control calibration prior to analysis by flow cytometry.

### Analysis

Flow cytometry was performed using a Cytek Northern Lights spectral cytometer equipped with a 488 nm laser and 14 fluorescent emission filters, and data were collected using SpectroFlo v3.0.0 software. Flow cytometry data processing was done using FlowJo v10. Statistical analysis was done using GraphPad Prism 9. All data are presented as mean ± SEM for 3 independent experiments. P-values were calculated after 1-way or 2-way ANOVA with Tukey or Dunnett post hoc tests depending on the experiment performed. *P* values < 0.05 were considered statistically significant. Experiment specific statistical analysis information can be found in the figure captions.

## Results

### Human monocytes phagocytose viable and heat-inactivated human MSCs in a dose-dependent manner

**Fig. 1:**
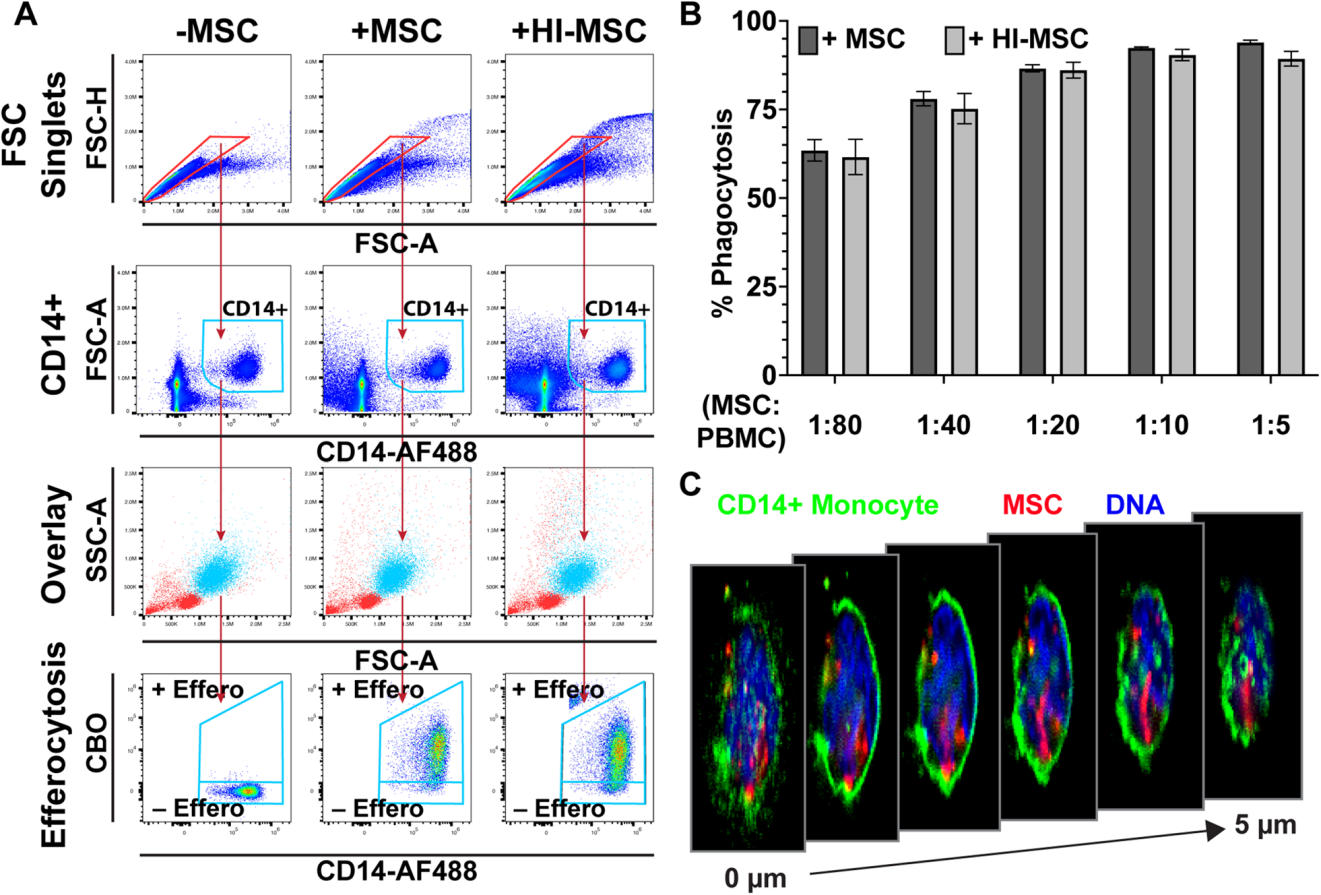
Human monocytes phagocytose viable and heat-inactivated human MSCs in a dose-dependent manner. **a** Gating strategy and representative plots of CD14 + monocytes alone (−MSC) or cultured with CBO-stained MSCs (+MSC) or HI-MSCs (+HI-MSC) for 24 h. **b** The percent of CD14 + monocytes positive for CBO increases with increasing dose of MSCs and HI-MSCs after 24 h of non-adherent co-culture (mean ± SEM, *n* = 3 independent experiments). **c** Representative Z-stack cross sections at 1 µm intervals of a CD14-AlexaFluor 488 stained monocyte (green) after 24 h coculture with CBO-stained MSCs (red). Nuclei stained blue with Hoechst33342

The hypothesis that efferocytosis of MSCs is responsible for MSCs immunosuppressive effects raises the question of whether the vitality of an MSC product is critical for therapeutic efficacy. Thus, we first wanted to determine if the phagocytic response of human peripheral monocytes differed upon encountering viable versus heat-inactivated MSCs (HI-MSCs). We cultured PBMCs with viable- or HI-MSCs for 24 h, after which phagocytosis was determined by the percent of CD14+ cells that were positive for CellBrite Orange (CBO), a membrane stain used to label MSCs prior to co-culture (Fig. 1a). We found viable and HI-MSCs were phagocytosed to equivalent amounts in a dose-dependent manner with the highest MSC:PBMC ratio exhibiting the most phagocytosis (Fig. 1b). Phagocytosis of MSCs by CD14 + human monocytes was confirmed by Z-stack imaging using an inverted fluorescent microscope which revealed intracellular depots of CBO cargo within CD14 + monocytes (Fig. 1c). This indicates that human MSCs are efficiently phagocytosed by human monocytes regardless of whether the cells have high or low viability or metabolic activity (Additional file 1: Figure S1).

### Human T-cell proliferation is suppressed following phagocytosis of MSCs in a dose-dependent manner

**Fig. 2:**
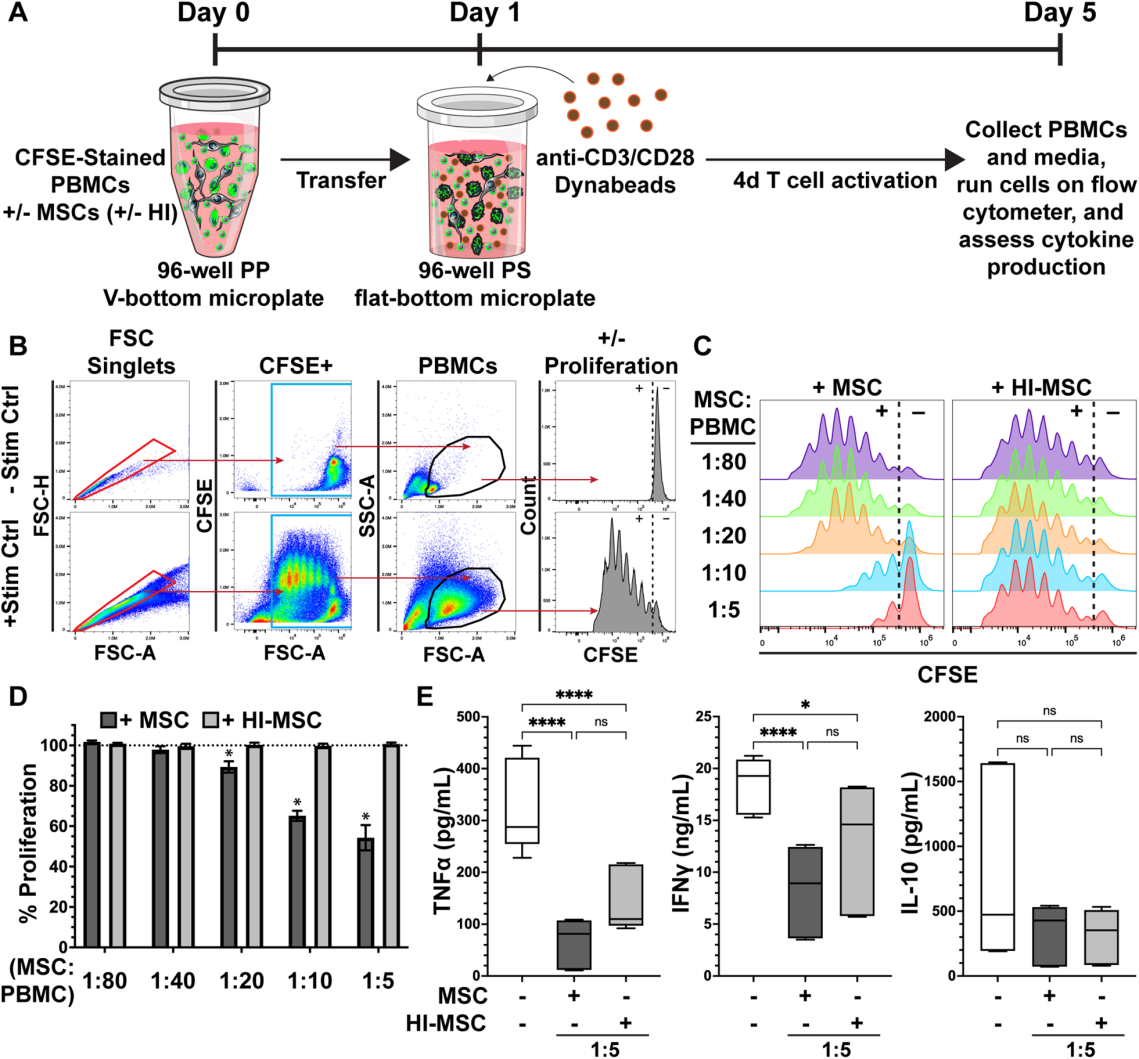
Human T-cell proliferation is suppressed following 24 h exposure to MSCs in a dose-dependent manner. **a** Schematic of 24 h efferocytic-licensing of monocytes in suspension followed by replating and 4d T-cell activation. **b** Gating strategy for T-cell proliferation analysis. After singlet discrimination, CFSE positive PBMCs were gated, and the T-cell population was gated using FSC-SSC. % proliferation was then calculated using a gate based on the No Stim Control. **c** Representative T-cell proliferation histograms for MSC and HI-MSC culture conditions at each MSC:PBMC ratio tested. The dotted line denotes the proliferative (+) from the non-proliferative (−) cells. **d** Quantification of T-cell proliferation as a percent of the positive stim control for each MSC:PBMC ratio. (Mean ± SEM, *n* = 3 independent experiments, *p < 0.05 calculated after 2-way ANOVA with Dunnett post hoc test to compare + MSC and + HI-MSC at each ratio to normalized stimulated control (dashed line)). **e** Quantification of pro- and anti-inflammatory cytokines detected after T-cell activation without or with efferocytic-licensing by viable or HI-MSC (box and whisker show 25th, 50th, and 75th percentile while whiskers show min and max values, *n* = 3 independent experiments, *p < 0.05 calculated after 1-way ANOVA with Tukey post hoc test to compare all conditions)

Having established that the health status of MSCs did not affect monocyte’s ability to phagocytose them, we next wanted to determine how phagocytosing MSCs impacted monocyte function. Specifically, we wanted to determine if phagocytosing MSCs resulted in the monocytes taking on an inflammatory or immunosuppressive phenotype. To test this, we again allowed monocytes to phagocytose either viable or HI-MSCs for 24 h and then transferred the PBMCs to a new plate to remove residual MSCs before T-cell activation (Fig. 2a, b). While T-cell proliferation was not changed after phagocytosis of HI-MSC, we found T-cell proliferation was suppressed in conditions with increasing amounts of viable MSCs that were present during the efferocytic-licensing (Fig. 2c, d). This data show that efferocytosis of viable, but not HI-MSCs, induces monocytes to take on an immunosuppressive phenotype toward activated T-cells that results in significant blunting of activated T-cell proliferation.

To further gauge how efferocytosis of MSCs causes monocytes to alter the local inflammatory environment in the setting of T-cell activation we assayed three key soluble immune factors, TNF-α, IFN-γ, and IL-10. Exposure of PBMCs to viable and HI-MSCs for 24 h to allow for efferocytosis followed by T-cell activation resulted in significant reduction in TNF-α and IFN-γ production compared to monocytes without efferocytic-licensing (Fig. 2e). For both TNF-α and IFN-γ, the reduction was largest when monocytes efferocytosed viable-rather than HI-MSC. Interestingly, while efferocytosis of viable MSC led to significant levels of T-cell suppression, we found no difference in the levels of IL-10 between HI-MSC and viable MSC, but both conditions resulted in a narrower range of IL-10 levels compared to the no-efferocytosis control (Fig. 2e). This data demonstrate that efferocytosis of viable- and HI-MSC by monocytes significantly alters the inflammatory milieu by reducing levels of inflammatory cytokines.

### Human T-cell proliferation is suppressed by MSC-licensed monocytes even after removal of residual MSCs

**Fig. 3:**
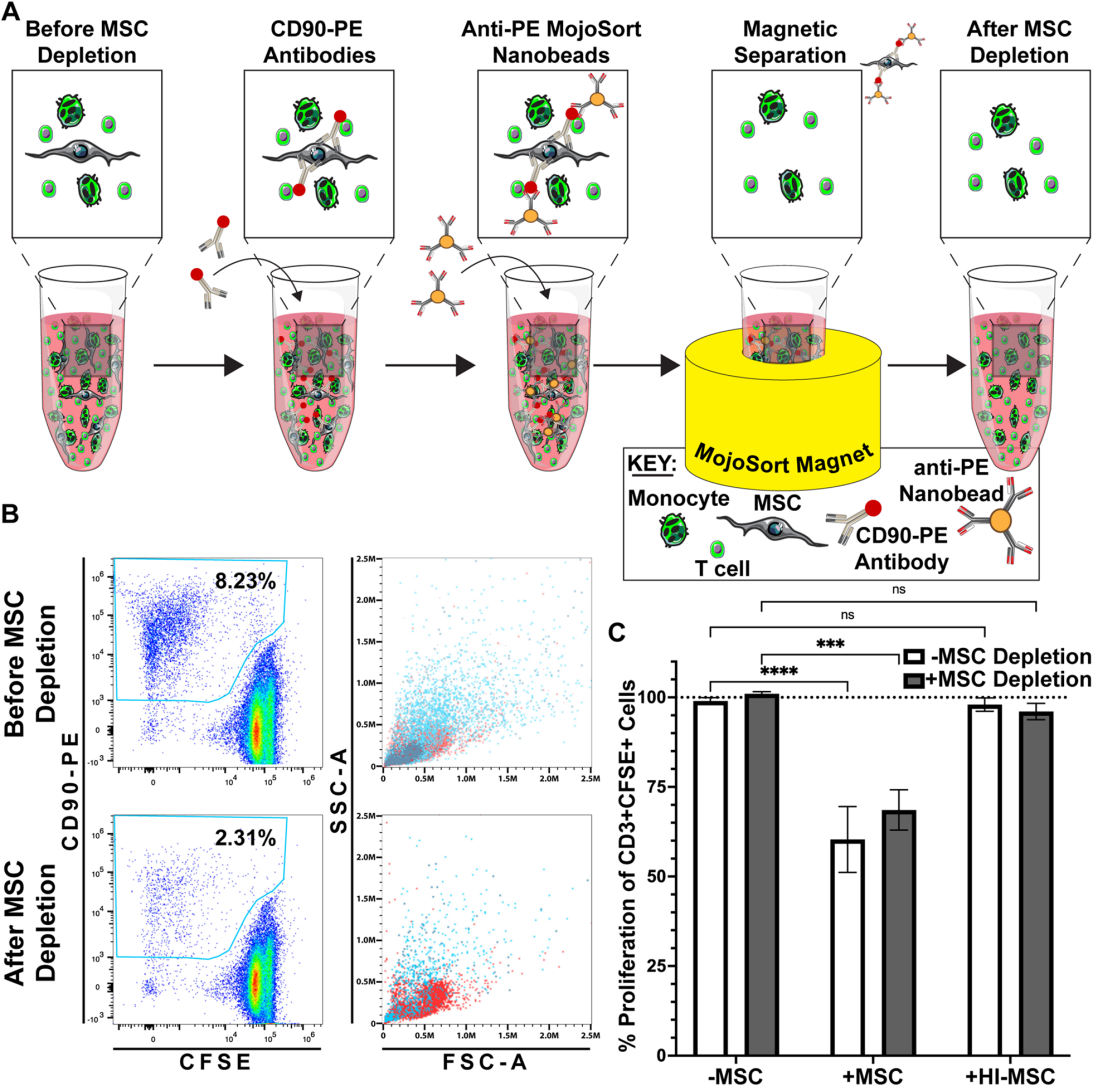
Human T-cell proliferation is suppressed by MSC-licensed monocytes even after residual MSCs are removed. **a** Schematic of process used to remove residual MSCs after 24 h MSC:PBMC coculture. Residual MSCs were labeled with a CD90 antibody conjugated to PE and then tagged with Anti-PE MojoSort nanobeads and pulled down with a magnet. **b** Representative plots showing efficiency of MSC removal. Left panels show gate for CD90 + MSCs and the right panels show an overlay of total cells (red) and CD90 + MSCs (light blue). **c** Quantification of T-cell proliferation as a percent of the positive stim control for 1:5 MSC:PBMC (mean ± SEM, *n* = 3 independent experiments, *p < 0.05 calculated after 2-way ANOVA with Tukey post hoc test)

Next, we wanted to definitively confirm that the suppression of T-cells we were observing was due to efferocytically-licensed monocytes, and not residual viable MSCs in the co-culture. While our initial experiments utilized a replating strategy to leave non-efferocytosed MSCs behind before T-cell activation, we noted small spheroids of non-phagocytosed viable MSCs were still transferred during the replating of cells from polypropylene V-bottom microplates to polystyrene flat-bottom microplates. By the end of the assay the residual MSCs had attached and spread out in the wells (Additional file 1: Figure S2). Previous work from our group has shown that spheroid MSCs are not immunosuppressive toward T-cells [17], but we wanted to confirm that the residual MSCs were not contributing to suppression. To determine if the suppression was due to monocytes that had efferocytosed MSCs or the non-efferocytosed residual MSCs, we depleted non-efferocytosed MSCs from our culture after the 24 h efferocytosis period, and then plated the T-cell activation assay (Fig. 3a). Using the magnetic bead-based depletion technique, we observed 70–80% reduction in MSCs (Additional file 1: Figure S3A, B) that reduced the effective MSC-to-PBMC ratio to ~ 1:50, well below what is needed for viable MSCs to impact T-cell proliferation (Fig. 3b). To verify that any remaining MSCs after depletion from the MSC:PBMC coculture were not suppressive on their own, different cell numbers of MSCs were plated followed by a fixed number of non-MSC educated PBMCs to achieve MSC-to-PBMC ratios of 1:5 to 1:80. Only co-cultures with MSC ratios of 1:10 or greater showed significant levels of T-cell suppression (Additional file 1: Figure S3B). Of note, even our passive MSC depletion technique relying on transfer of PBMCs from v-bottom polypropylene plates after the efferocytosis period reduces the amount of residual MSCs to a ratio of 1:14. Thus, this quality control assay confirms that the small amount of residual MSCs after depletion is not responsible for the T-cell suppression observed after efferocytosis of viable MSC. After the depletion of non-efferocytosed MSCs we observed that monocytes that have efferocytosed viable MSCs significantly suppress T-cell proliferation (Fig. 3c). This suggests efferocytosis of viable MSCs by monocytes is critical for monocytes to take on an immunosuppressive phenotype toward activated T-cells.

### Human monocytes experience differential phenotypic shift upon efferocytosis of viable-versus HI-MSC

**Fig. 4:**
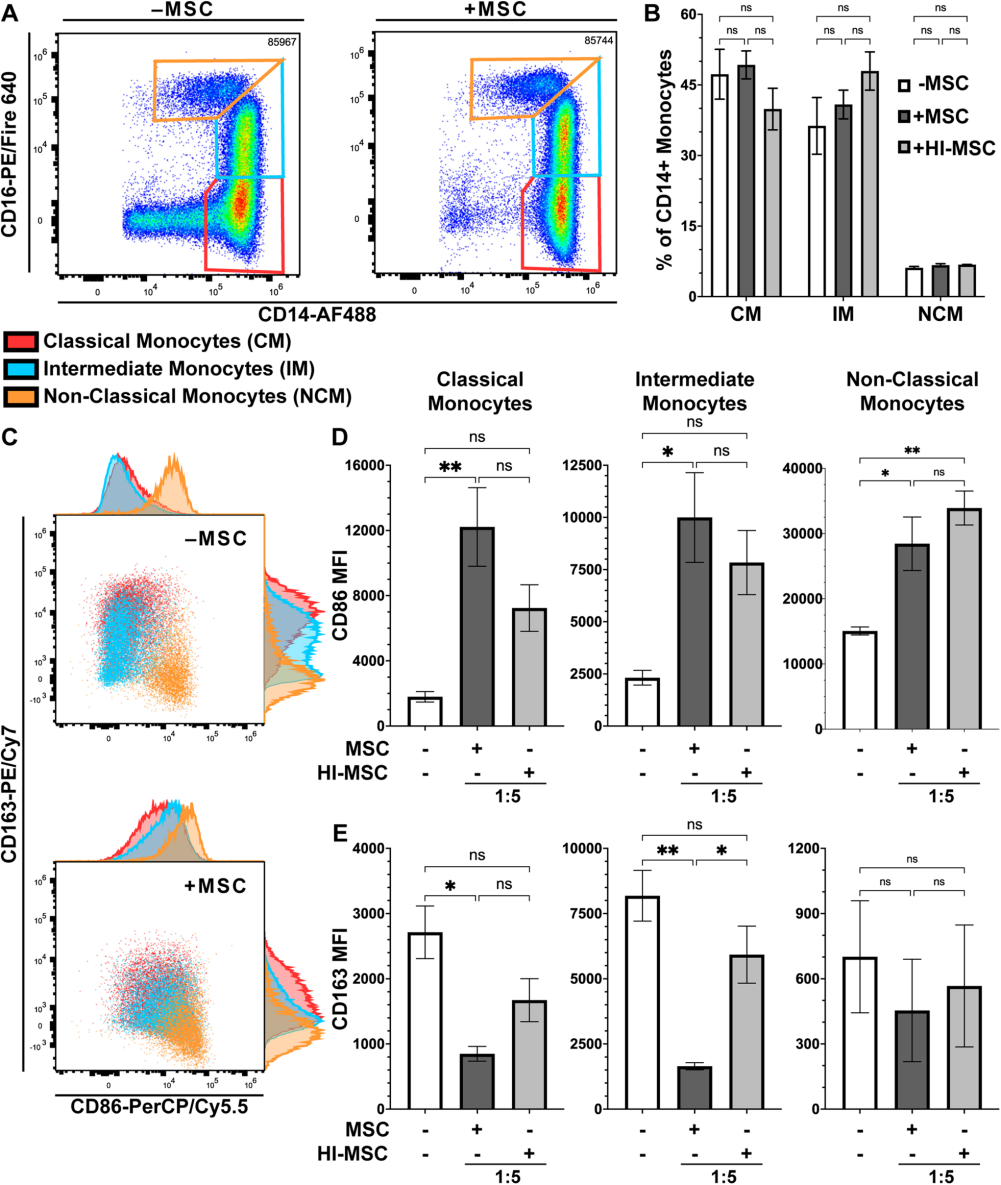
Human monocytes experience differential phenotypic shift upon efferocytosis of viable- versus HI-MSC. **a** Representative plots of CD14 versus CD16 of CD14 + monocytes after 24 h of efferocytosis of MSCs. Red gate = classical monocytes (CM), Blue gate = intermediate monocytes (IM), and Orange gate = non-classical monocytes (NCM). **b** Quantification of monocytes identified as CM, IM, or NCM (mean ± SEM, *n* = 3 independent experiments, *p < 0.05 calculated after 2-way ANOVA with Tukey post hoc test). **c** CD86 versus CD163 representative plots of CM (red), IM (blue), and NCM (orange) subsets overlaid and associated histograms. **d** Quantification of CD86 MFI for CM, IM, and NCM. (Mean ± SEM, *n* = 3 independent experiments, *p < 0.05 calculated after 1-way ANOVA with Tukey post hoc test). **e** Quantification of CD163 MFI for CM, IM, and NCM. For the gating strategy, refer to Additional file 1: Figure S4. (Mean ± SEM, *n* = 3 independent experiments, *p < 0.05 calculated after 1-way ANOVA with Tukey post hoc test)

Next, having established that the efferocytically-licensed monocytes were suppressive toward T-cells, we wanted to determine how the monocytes were changing phenotypically. Specifically, we wanted to determine how monocyte subsets as well as their surface marker inflammatory profiles change using a panel of five surface markers: CD14 and CD16 for determining monocyte subsets, CD86 as a traditional inflammatory marker for M1-like monocytes, and CD163 and CD206 as traditional anti-inflammatory markers for M2-like monocytes (Additional file 1: Figure S4). After culture with either viable or HI-MSCs, monocytes do not significantly alter their CD14 versus CD16 profile and thus the fraction of monocytes belonging to classical (CD14^hi^CD16-), intermediate (CD14^hi^CD16^lo/int^), or non-classical subsets (CD14^int^CD16^hi^) is relatively unchanged by efferocytosis (Fig. 4a, b). However, there is a significant shift in CD14^lo^CD16-cells into the various monocyte subsets when MSCs are a part of the culture system (Fig. 4a). Monocytes efferocytically-licensed with MSCs and HI-MSCs increase their CD86 expression and decrease their CD163 expression for each monocyte subset compared to control, but viable MSCs induce the greatest changes (Fig. 4c). CD86 expression is significantly increased in classical and intermediate monocytes for viable MSC educated monocytes compared to control (Fig. 4d). For non-classical monocytes CD86 expression is significantly increased for both viable and HI-MSC efferocytically-licensed monocytes compared to control (Fig. 4d). CD163 expression is significantly reduced in classical and intermediate monocytes for viable MSC educated monocytes compared to control (Fig. 4e). To our surprise, we were not able to detect significant levels of CD206 for any monocytes with or without efferocytosis of MSCs (Additional file 1: Figure S5). From this 5-color panel, we see that the surface marker profile of viable MSC efferocytically-licensed monocytes, which suppress T-cell proliferation, adopt a predominantly CD86^hi^CD163^lo^ phenotype while non-suppressive HI-MSC efferocytically-licensed monocytes, adopt a predominantly CD86^int^CD163^int^ phenotype.

### Efferocytosis of viable but not HI-MSCs by monocytes induces secretion of IL-10 and activation of IDO

**Fig. 5:**
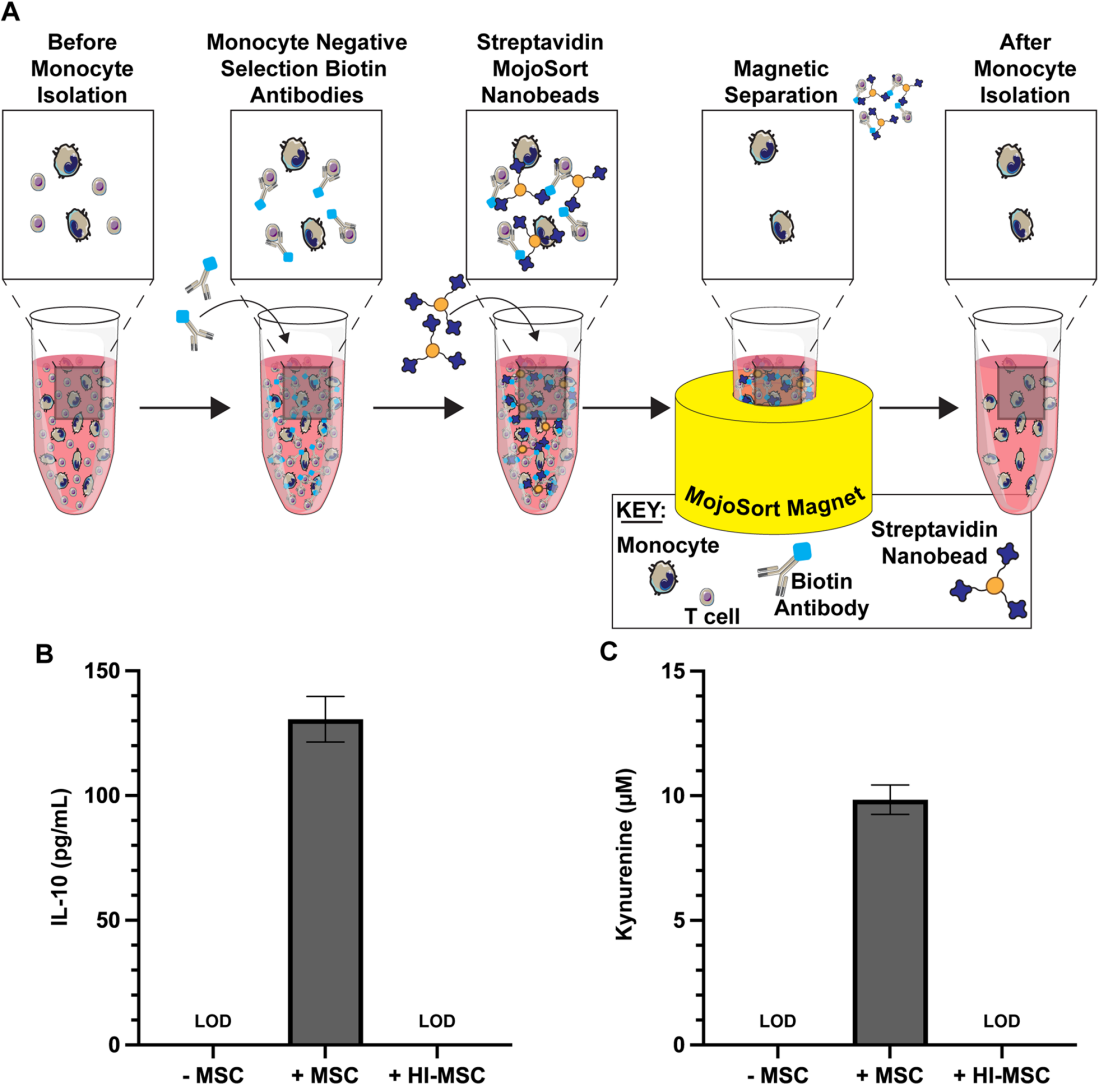
Efferocytosis of viable but not HI-MSCs by monocytes induces IL-10 secretion and activation of IDO. **a** Schematic of process used to negatively select monocytes. Non-monocytes were labeled with a cocktail of biotin-conjugated antibodies, then tagged with streptavidin MojoSort nanobeads, and pulled down with a magnet. Quantification of **b** IL-10 and **c** kynurenine production detected after 2-day monocyte culture without or with efferocytic-licensing by viable or HI-MSCs. LOD = Limit of Detection (LOD_IL-10_ = 19 pg/mL, LOD_Kyn_ = 7 µM, LOD_TNF-α_ = 15 pg/mL (data not shown))

To further gauge the functional phenotype of monocytes following efferocytosis of MSCs, we assayed monocytes secretory profile in the absence of T-cells (Fig. 5a). Isolated monocytes were cultured with viable or HI-MSCs for 24 h after which the samples were transferred to a new plate with new media. The monocytes were cultured for 2 additional days after which the media was assayed for IL-10, kynurenine, and TNF-α. Monocytes that efferocytosed viable MSCs showed large increases in IL-10 (Fig. 5b) and kynurenine (Fig. 5c) production compared to control and HI-MSC educated monocytes. Control and HI-MSC educated monocytes did not reach the limit of detection (LOD_IL-10_ = 19 pg/mL, LOD_Kyn_ = 7 µM). TNF-α was below the limit of detection (LOD_TNF-α_ = 15 pg/mL) for all conditions tested (data not shown). Notably, levels of IL-10, kynurenine, and TNF-α were all below the limit of detection for additional quality controls that included viable or HI-MSCs without monocytes (data not shown). Together, this data suggest that monocytes that have efferocytosed viable MSCs exhibit an anti-inflammatory secretory profile while HI-MSC educated monocytes behave similarly to naïve monocytes.

### Human monocytes experience differential secretome shift after simulated sepsis stimulation depending on if they efferocytosed viable versus HI-MSC

**Fig. 6:**
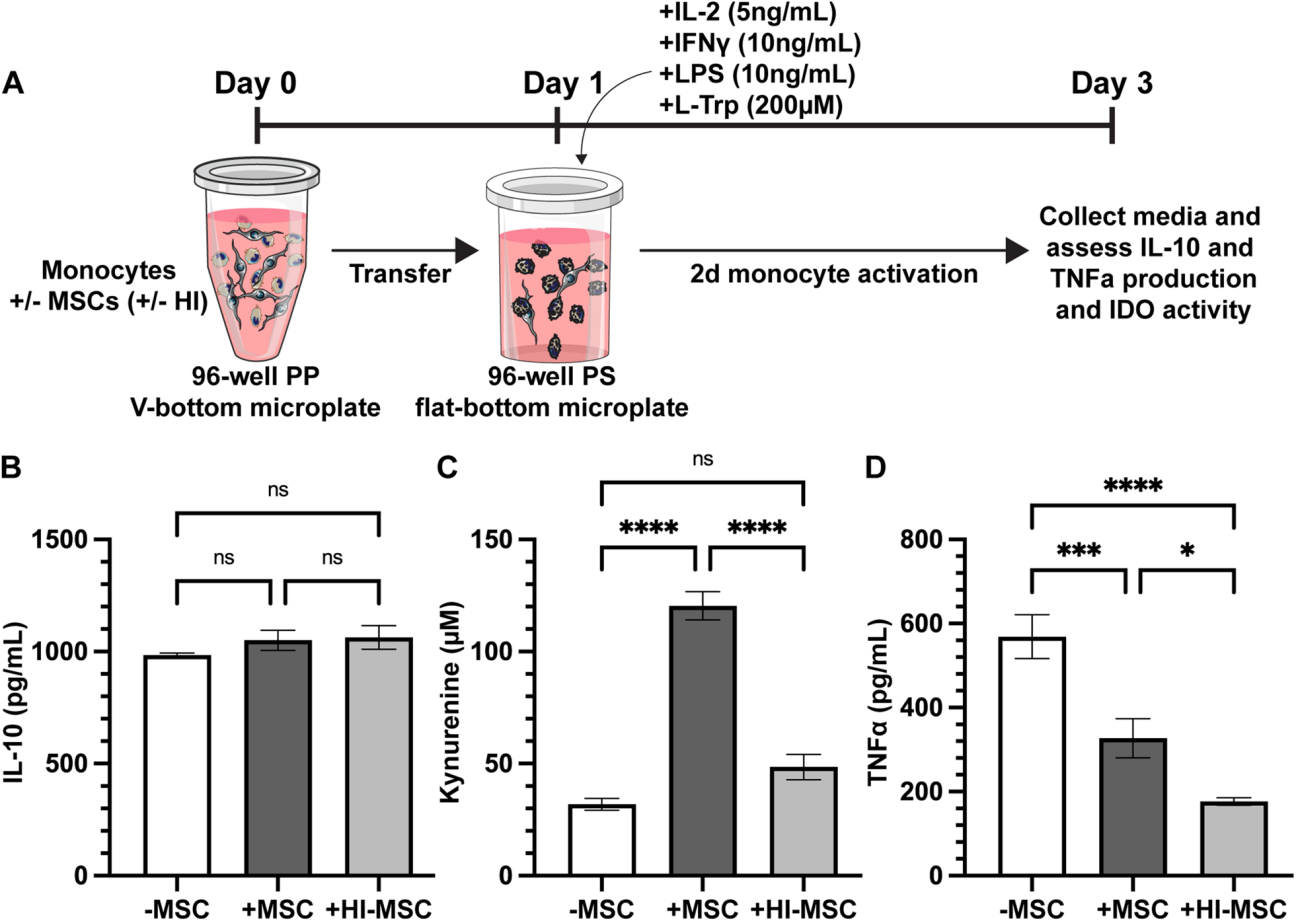
Monocytes experience differential secretome shift after simulated sepsis stimulation dependent on MSC efferocytosis condition. **a** Schematic of isolated human monocyte efferocytic-licensing without and with viable- or HI-MSCs followed by simulated sepsis stimulation with IL-2, IFN-γ, and LPS. See Fig. 5a for monocyte isolation process. Quantification of **b** IL-10, **c** kynurenine, and **d** TNF-α production detected after 2-day sepsis stimulation without or with efferocytic-licensing with viable- or HI-MSCs (mean ± SEM, *n* = 3 independent experiments, *p < 0.05 calculated after 1-way ANOVA with Tukey post hoc test)

Having established that efferocytosis increases the expression of immunosuppressive factors in naïve monocytes, we next wanted to determine if it impacted their immunoregulatory profile once stimulated with inflammatory mediators. Since viable and HI-MSCs have been shown to have efficacy in vivo in sepsis models [33, 49], we sought to simulate the activation monocytes see in the setting of sepsis. To mimic the inflammatory environment of sepsis, we added LPS, IFN-γ, and IL-2 during the 2-day isolated monocyte culture (Fig. 6a). After 2 days of activation, we observed very high levels of IL-10 with and without efferocytosis, but none of the conditions were significantly different from each other (Fig. 6b). Kynurenine production due to efferocytosis of viable MSCs was significantly increased compared to monocytes alone and monocytes that efferocytosed HI-MSCs (Fig. 6c). Interestingly, monocytes that efferocytosed either viable or HI-MSCs resulted in significant reduction in TNF-α compared to monocytes alone, but the monocytes that had efferocytosed HI-MSC had significantly less TNF-α compared to those that efferocytosed viable MSCs (Fig. 6d). These results suggest that efferocytosis of HI-MSCs by monocytes results in the monocytes adopting an immune-resolving profile within septic inflammatory environments and help explain prior observations that HI-MSC may be superior to viable MSC in the treatment of sepsis [33, 49].

## Discussion

The journey toward widespread clinical use of MSCs has been slower and more challenging than most researchers and patients have hoped. Despite encouraging performance in numerous animal models and positive data in clinical trials, the approval of MSC therapies has been hampered by a lack of a complete understanding of MSCs mechanism of action. Without clearly defined mechanisms of action that correlate with clinical efficacy, the quality of MSCs from lot to lot cannot be verified. MSCs clearly exert immunosuppressive actions toward T-cells and monocytes via trophic factors, and these factors can be readily assessed in potency assays [10, 48, 51–53]. But are these the only, or even primary mechanisms of action at work in vivo? The limited in vivo persistence of MSCs coupled with the observed durability of MSC’s immunosuppressive effects suggest MSCs leave a lasting imprint on the host immune system, but how exactly?

Recently, efferocytosis has emerged as a potential mechanism of action exerted by MSCs on the host immune system that helps explain the durable effects of MSCs. To date, several groups have shown monocytes and macrophages uptake MSCs and this uptake is correlated with an anti-inflammatory profile [8, 18, 30, 32, 39, 54, 55]. Pang et al. [30] showed that apoptosis-resistant MSCs delayed onset of symptoms in an EAE model, but ultimately performed worse than non-resistant MSCs, suggesting apoptosis is important for the long-term immunosuppressive effects of MSCs. Meanwhile, Cheung et al. induced MSC apoptosis using an anti-Fas monoclonal antibody and found monocytes uptake the dying cells and take on an immunosuppressive phenotype [18]. While these studies provide evidence that efferocytosis is a potential mechanism, it raises the question, does the viability of MSC’s even matter and how robust is monocyte-mediated immunosuppression once the MSCs are removed? Answers to these questions have been mixed [56], with some studies showing viable MSCs are essential while others showing comparable or even superior effects when administering heat-inactivated MSCs (HI-MSCs). For example, viable MSCs prolonged lifespan of mice receiving allogeneic heart transplants compared to HI-MSCs [33] while in a sepsis model HI-MSCs significantly prolonged lifespan of mice compared to viable MSCs [33, 49]. This indicates that the condition of the MSCs at the time of administration has different therapeutic effects depending on the disease in question. In these in vivo studies, MSCs are delivered systemically and can, therefore, contribute both efferocytic and non-efferocytic mechanisms. Because both mechanisms are potentially active, it is difficult to tease apart which mechanism is directing immune modulation.

Here we took advantage of the flexibility and control afforded by in vitro systems to address these questions by using a variety of cell separation techniques to remove non-phagocytosed MSCs and isolate specific immune subsets. These techniques ranged from replating monocytes after efferocytosis in new wells to using antibody-conjugated magnetic beads to remove undesired cell populations and isolate our focus on the phenotype and function of monocytes after efferocytosis. In contrast to previous reports [18] that showed prior induction of apoptosis was essential for uptake by monocytes, we found human monocytes efficiently take up MSCs in a dose-dependent manner regardless of if they are viable or heat inactivated (Fig. 1). We saw very high levels of uptake at even modest ratios, with > 75% of monocytes uptaking MSCs in both conditions at an MSC:PBMC ratio of 1:40 (Fig. 1). In addition, we saw similarly efficient uptake even when the monocytes were first isolated from the PBMC population (Additional file 1: Figure S6), suggesting killing by cytotoxic T-cells is not required for monocytes to efferocytose MSCs. Importantly, in our studies we allowed efferocytosis to occur in non-adherent V-bottom plates to mimic the non-adherent conditions MSCs encounter in circulation with immune cells.

Having established that naïve primary human monocytes efficiently uptake both viable and HI-MSC, we next evaluated the monocytes through a series of assays to determine their immunologic profile. We found that uptake of viable-but not HI-MSC led monocytes to suppress T-cell activation (Fig. 2). While efferocytosis of viable MSCs by monocytes led to statistically significant T-cell suppression at MSC:PBMC ratios of 1:20, efferocytosis of HI-MSC never resulted in T-cell suppression. These observations agree with prior studies that assessed viable and HI-MSC effects on suppression of T-cells [33, 49]. Recognizing that the suppression observed in our study and those reported previously could be due to residual viable MSCs in the co-culture, we repeated the experiment with a novel additional step to remove residual MSCs using an anti-CD90 antibody depletion technique. Even after removal of residual MSCs, our observations still held showing that the efferocytically-licensed monocytes are responsible for the suppression of T-cells (Fig. 3, Additional file 1: Figure S3). This provides some of the most direct evidence to date that efferocytic-licensing of monocytes leads to monocytes taking on an immunosuppressive phenotype toward T-cells in the absence of residual viable MSC. It also highlights that the quality of the MSC material has a direct impact on the functional phenotype of the monocyte after efferocytosis.

Interestingly, efferocytosis of both viable- and HI-MSC resulted in significant reduction in the inflammatory cytokines TNF-α and IFN-γ (Fig. 2E). Thus, we expanded our characterization beyond T-cell suppression by looking at surface marker expression. While it had been previously reported that efferocytosis leads to an increase in intermediate monocytes [39], in our hands we found only a modest non-significant increase in intermediate monocytes (Fig. 4a, b). While methodologies were similar, a key difference likely explains the discrepancy; while we analyzed monocyte surface markers changes after efferocytosis within complete PBMC cultures untouched by selection antibodies, previous studies used monocytes isolated using positive selection with a CD14 antibody [39]. Positive selection of monocytes with anti-CD14 antibodies has been shown to significantly skew monocyte and monocyte-derived macrophage phenotype [57, 58]. While we did not see shifts in CD14 and CD16, we did see significant shifts in the levels of both CD86 and CD163 (Fig. 4c). CD86 significantly increased in all monocytes after efferocytosis of viable MSC but only significantly in non-classical monocytes after efferocytosis of HI-MSC (Fig. 4d). The increase in CD86 was a surprise because it is traditionally considered a pro-inflammatory marker for M1 macrophages since it helps stimulate T-cell activation when it interacts with CD28; notably, however, CD86 also interacts with CTLA-4 to halt T-cell activation*.* While we did not explore the role of CD86 further in this study, CD86 could be a marker of interest for future investigation as it has dual pro-inflammatory and anti-inflammatory roles. CD163, on the other hand, significantly dropped in classical and intermediate monocytes only after efferocytosis of viable MSC (Fig. 4e). Previous studies have reported an increase in both CD163 and CD206 [39], which is in contrast to what we observed, again likely due to our use of untouched monocytes rather than anti-CD14 positively selected monocytes. We consistently observed a decrease in CD163 and no detectable CD206 above isotype control after efferocytosis of viable MSC. Collectively, this surface marker data show that monocytes take on divergent phenotypes depending on if they efferocytose viable- or HI-MSC, but the surface marker profiles both trend toward pro-inflammatory.

To explore the monocyte immunological landscape further, we assayed the secretion profile of viable and HI-MSC efferocytically-licensed monocytes both without and with a subsequent inflammatory stimulus designed to mimic sepsis. We observed that isolated naïve monocytes efferocytically-licensed with viable but not HI-MSCs had increased production of IL-10 and kynurenine and no detectable production of TNF-α (Fig. 5). This data showed that efferocytosis of viable MSCs by monocytes does indeed lead to an immune-resolving phenotype as evidenced by increased T-cell suppression and an increase in the production and activity of anti-inflammatory IL-10 and IDO, respectively. When the efferocytically-licensed monocytes were subsequently challenged with a cocktail of LPS, IFN-γ, and IL-2 to simulate sepsis, we saw IL-10 increase in all samples, but only monocytes efferocytically-licensed with viable MSC significantly increased output of kynurenine (Fig. 6). Both viable and HI-MSC efferocytically-licensed monocyte exhibited a significant reduction in TNF-α secretion, but, notably, the HI-MSC licensed monocytes had significantly more reduction in TNF-α than the viable MSC licensed monocytes. Prior studies have shown a similar reduction in TNF-α secretion, except that they had shown that treatment with viable MSCs tends to reduce TNF-α secretion more than HI-MSC [33, 49]. Importantly, in our study the monocytes were isolated using a negative selection kit and were re-plated after efferocytic-licensing to remove the majority of residual viable MSCs. In addition, we simulated sepsis using a cocktail of LPS, IFN-γ, and IL-2, whereas the prior studies used LPS alone.

It is clear that monocytes adopt distinctly immunosuppressive functionality in different inflammatory environments dependent on the type of MSC material they efferocytose; however, the overall phenotypic profile of the monocytes is complex. Surface marker characterization suggests relatively pro-inflammatory profiles for viable and HI-MSC efferocytically-licensed monocytes, yet secretory profiling suggests strong anti-inflammatory characteristics. These results reveal that monocytes adopt a mixed M1/M2 intermediate profile. Other studies of monocytes [59, 60], monocyte-derived dendritic cells [61, 62], and monocyte-derived macrophages [59, 60] have shown results consistent with ours regarding increased CD86 expression, IL-10 secretion, IDO activity, and decreased TNF-α secretion [59]. For example, Han et al. analyzed circulating CD14 + , CD86 + dendritic cells from human hepatocellular carcinoma patients and found they express high levels of IL-10 and IDO and were capable of suppressing T-cell proliferation [62]. Similarly, Zahorchak et al. found monocytes from peripheral blood could take on a CD86 + profile while producing high levels of IL-10 and low levels of TNF-α [61]. In vivo, monocytes are highly plastic cells that are very sensitive to stimuli and, thus, rarely partition strictly into M1 or M2 phenotypes, but rather fall on a spectrum from pro-inflammatory to anti-inflammatory [63–66].

While this work adds to our understanding of MSC efferocytosis, several major questions remain. Studies to date on efferocytosis, including the present one, analyzed efferocytosis using naïve primary monocytes from healthy donors. If efferocytosis is a major mechanism of action for MSC therapies, how do host-factors influence the response to therapy? We know even co-morbid disease states like obesity dramatically alter the immune system [4], but how will they impact efferocytosis? In vivo, multiple mechanisms of action ranging from anoikis [67], to cytotoxic T-cell mediated lysis [68], to complement-mediated mechanisms can lead to MSC apoptosis [1, 69], but how the mechanism of apoptosis influences the efferocytic response of monocytes is yet to be determined within the context of MSC therapy. Furthermore, in the present study we compared viable and HI-MSC, but the variety of MSC products available today is highly diverse. MSCs are being manufactured using different culture systems, tissues of origin, and priming strategies [7], each of which could impact the efferocytic response. Finally, the discovery of efferocytosis as a mechanism does not rule out trophic factors as key mechanisms of action of MSC. Rather, it highlights that MSC therapies offer multiple mechanisms of action, and it is critical to understand which mechanisms are essential for specific disease indications [33].

## Conclusions

**Fig. 7:**
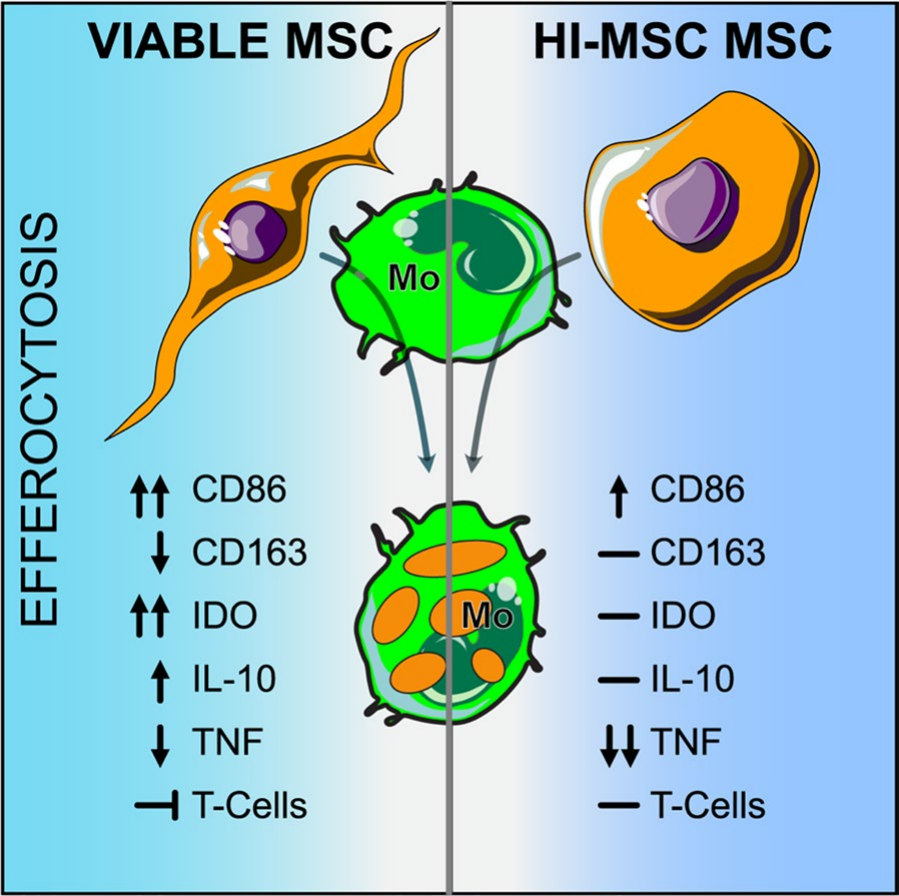
Monocyte phenotype and function depends on the initial viability of MSCs they efferocytose. Efferocytosis of viable MSC generated monocytes with increased expression of CD86, IDO activity, and immunosuppression toward T-cells. Efferocytosis of HI-MSC generated monocytes capable of decreasing total TNF-α production in simulated sepsis. This work highlights the importance of MSC quality, even for efferocytic mechanisms

Our understanding of MSC therapy has come a long way, from theories that MSCs are immune privileged, to the recognition that MSCs can be detected and cleared by the immune system [70], to the discovery that MSC clearance through efferocytosis is actually a critical aspect of their mechanism of action [71]. The current work used carefully designed in vitro studies to isolate monocyte efferocytosis of MSCs and provides evidence that efferocytosis of MSCs is a potent mechanism of action (Fig. 7), something that is difficult to tease apart in in vivo experiments. Additionally, the type of MSC material administered has considerable implications dependent on inflammatory states as we show with viable versus HI-MSC efferocytic-licensing conditions. The reality is likely that each disease offers a unique immunological challenge that requires different MSC mechanisms of action, and there is not a single silver bullet mechanism to explain all the effects of MSC-based therapies. The discovery of efferocytosis as a mechanism does not rule out trophic factors as key mechanisms of action of MSC. Rather, it highlights that MSC therapies offer multiple mechanisms of action, and it is critical to understand which mechanisms are essential for specific disease indications. Therefore, it is imperative that we continue to explore the numerous ways in which MSCs interact with immune cells to evaluate their potency, customize MSC therapies for specific disease applications, and identify patients most likely to benefit.

### Supplementary Information

**Additional file 1. Figure S1:** Heat-inactivation sets MSCs on a path toward apoptosis (related to Figure 1). **Figure S2:** Residual Non-phagocytosed viable MSC spheroids were transferred from 24hr efferocytic-licensing to T-cell activation plates (related to Figure 2). **Figure S3:** MSC depletion efficiency and dose-dependent immunomodulatory potency of healthy adherent MSC toward activated T-cells (related to Figure 3). **Figure S4:** Gating strategy for surface marker analysis of monocytes following MSC efferocytosis. **Figure S5:** CD206 expression of CD14+ monocytes without and with viable MSC efferocytic-licensing (related to Figure 4). **Figure S6:** Monocyte isolation is efficient and isolated monocytes phagocytose viable and HI-MSCs in the absence of Tcells (related to Figure 1).

## Abbreviations

ANOVA: Analysis of variance
Anti-Fas: CD95L, i.e., CD178
BCL-2: B-cell lymphoma 2
BSA: Bovine serum albumin
CBO: CellBrite Orange
CFSE: Carboxyfluorescein succinimidyl ester
CM: Classical monocyte
CTLA-4: Cytotoxic T-lymphocyte-associated protein 4, i.e., CD152
DI: Deionized
DMSO: Dimethyl sulfoxide
Dynabeads: Anti-CD3/CD28 T cell activation dynabeads
EAE: Experimental autoimmune encephalomyelitis
EDTA: Ethylenediaminetetraacetic acid
ELISA: Enzyme linked immunosorbent assay
FBS: Fetal bovine serum
Fc: Fragment crystallizable antibody region
FSC: Forward-scatter
GvHD: Graft-versus-host disease
HI: Heat-inactivated/inactivation
Hi: High expression
HLA: Human leukocyte antigen
HRP: Horseradish peroxidase
IDO: Indoleamine 2,3-dioxygenase
IFN-γ: Interferon γ
IL-10: Interleukin 10
IL-2: Interleukin 2
IM: Intermediate monocyte
Int: Intermediate expression
Kyn: Kynurenine
Lo: Low expression
LOD: Limit of detection
LPS: Lipopolysaccharide
LRC: Leukocyte reduction cone
M1: Pro-inflammatory monocyte/macrophage
M2: Anti-inflammatory monocyte/macrophage
MEM-α: Minimum Essential Medium α
MLR: Mixed lymphocyte reaction
MSC: Mesenchymal stem/stromal cell
NCM: Non-classical monocyte
PBMC: Peripheral blood mononuclear cell
PBS: Phosphate-buffered saline
PGE-2: Prostaglandin E2
RBC: Red blood cell
RPMI: Roswell Park Memorial Institute medium
SEM: Standard error of the mean
SSC: Side-scatter
TCA: Trichloroacetic acid
TMB: 3,3′,5,5′-Tetramethylbenzidine
TNF-α: Tumor necrosis factor α
TSG-6: Tumor necrosis factor-stimulated gene 6

## Declarations

### Availability of data and materials

All data are presented in the paper within the main and supplemental figures. Raw or unprocessed forms of the data are available upon request via email to james-ankrum@uiowa.edu.
