# Supplementary material for: Efferocytosis of viable versus heat-inactivated MSC induces human monocytes to distinct immunosuppressive phenotypes
Source: Stem Cell Res Ther. 2023 Aug 17;14:206. doi: 10.1186/s13287-023-03443-z (PMC10433682; doi:10.1186/s13287-023-03443-z)
Supplement: Supplementary file 1 — Additional file 1. Figure S1: Heat-inactivation sets MSCs on a path toward apoptosis (related to Figure 1). Figure S2: Residual Non-phagocytosed viable MSC spheroids were transferred from 24hr efferocytic-licensing to T-cell activation plates (related to Figure 2). Figure S3: MSC depletion efficiency and dose-dependent immunomodulatory potency of healthy adherent MSC toward activated T-cells (related to Figure 3). Figure S4: Gating strategy for surface marker analysis of monocytes following MSC efferocytosis. Figure S5: CD206 expression of CD14+ monocytes without and with viable MSC efferocytic-licensing (related to Figure 4). Figure S6: Monocyte isolation is efficient and isolated monocytes phagocytose viable and HI-MSCs in the absence of Tcells (related to Figure 1). [file 13287_2023_3443_MOESM1_ESM.docx]

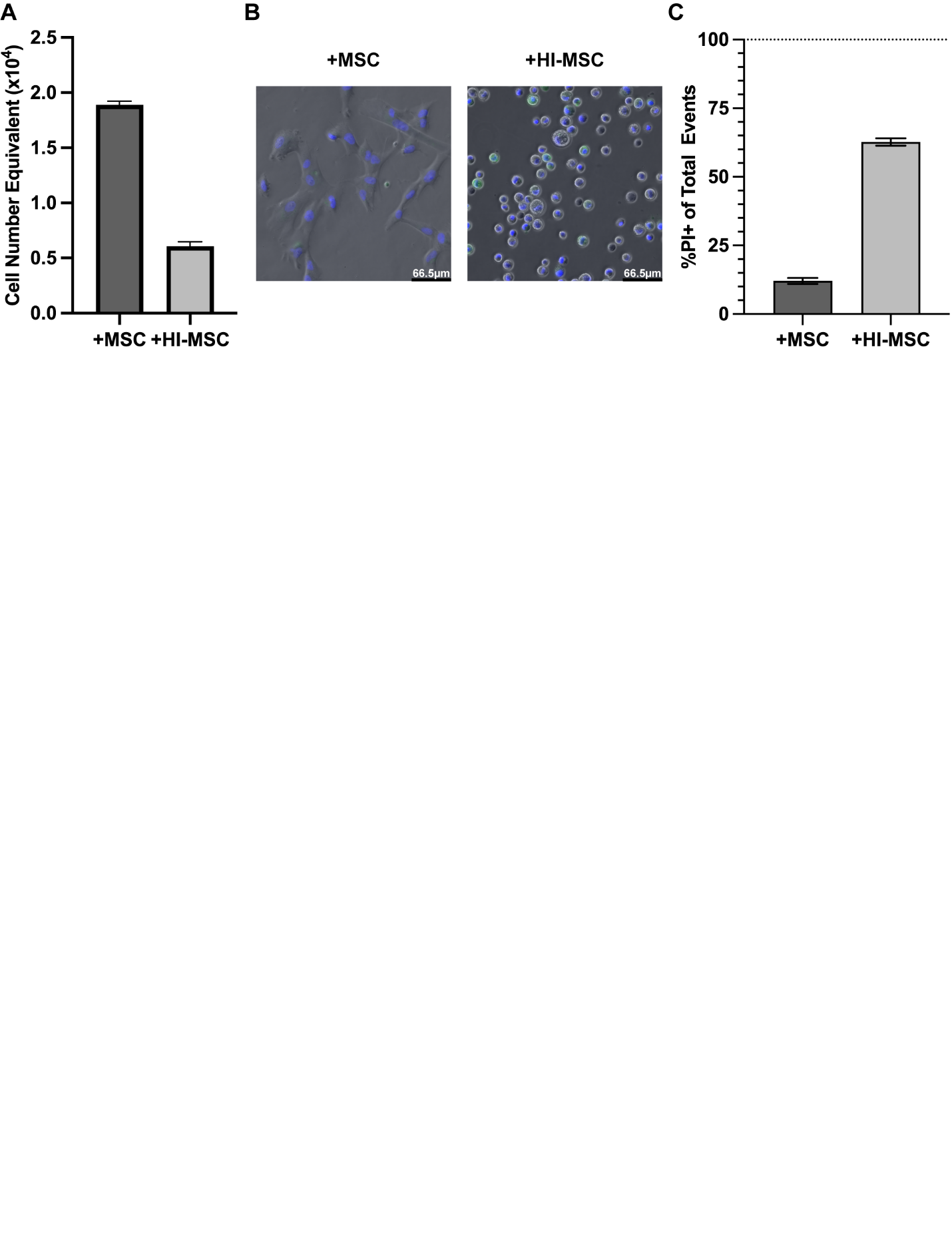


Figure S1: Heat-inactivation sets MSCs on a path toward apoptosis (related to Figure 1). (A) XTT assay of MSC vs HI-MSC showing cell number equivalent compared to standard cell count controls immediately after heat inactivation (Mean±SD, n=3 technical replicates. Cells were then plated and stained and imaged 24 hours later. (B) Fluorescent imaging showing 100% of HI-MSC 24hr post-plating remain balled-up and unattached. Blue = NucBlue nuclear stain (Invitrogen, Waltham, MA, Cat# R37605), Green = CellBrite Green membrane stain (Biotium, Fremont, CA, Cat# 30021). (C) Propidium iodide (PI) (Invitrogen, Waltham, MA, Cat# P3566) staining of MSC and HI-MSC 24hr after plating shows an increase in PI positive cell events with heat-inactivation (Mean±SD, n=3).


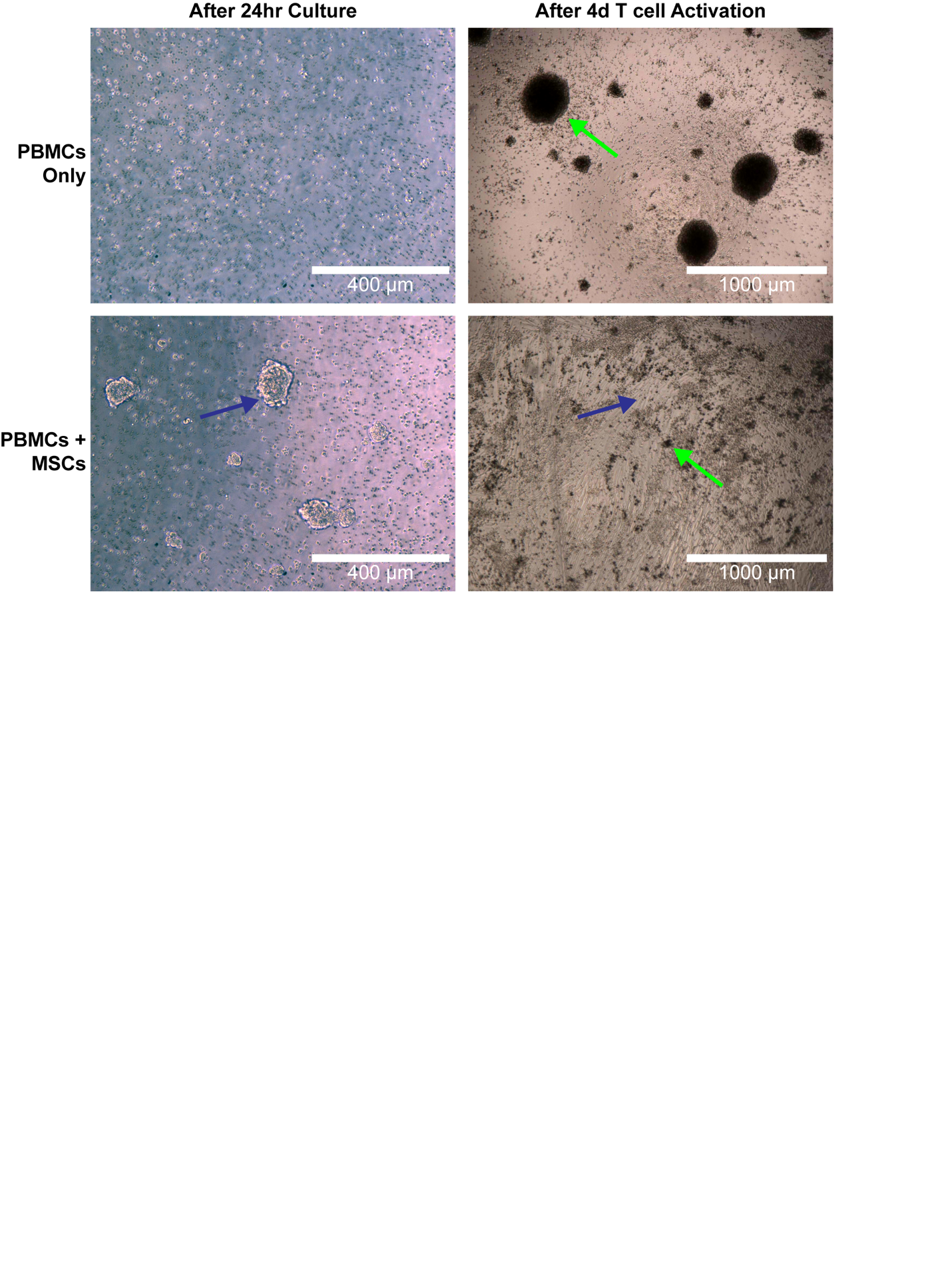


Figure S2: Residual Non-phagocytosed viable MSC spheroids were transferred from 24hr efferocytic-licensing to T-cell activation plates (related to Figure 2). After 4 days of culture, residual spheroid MSCs spread on plate. Green arrows = T-cells, Dark blue arrows = MSCs.


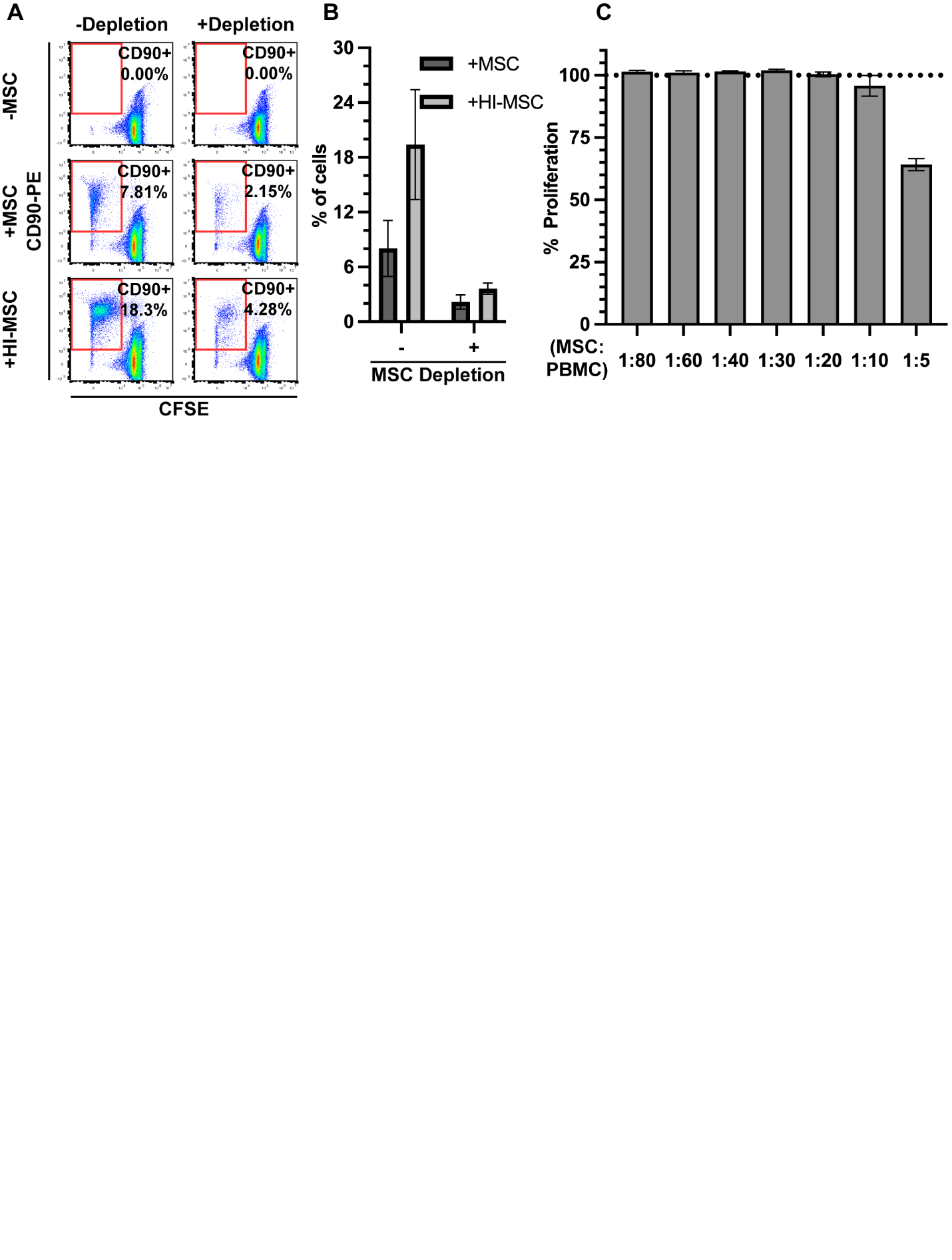


Figure S3: MSC depletion efficiency and dose-dependent immunomodulatory potency of healthy adherent MSC toward activated T-cells (related to Figure 3). (A) Anti-PE nanobead selection of CD90-PE labeled viable and HI-MSCs allows for efficient depletion of non-efferocytosed MSCs. MSC and HI-MSC were cultured in V-bottom polypropylene plates at a 1:5 MSC:PBMC ratio for 24 hours before collection and staining. (B) Without depletion, using the transfer method we see the ratio of MSC:PBMC drop from 1:5 to 1:14 (7%). With the nanobead depletion step residual MSCs drop to a ratio of ~1:50 (2%) MSC:PBMC. (Mean±SD, n=3). (C) When MSCs are not allowed to be efferocytosed ahead of T-cell activation, residual MSCs are not able to suppress T-cell proliferation at MSC:PBMC ratios observed during efferocytosis experiments. Results are normalized to positive stimulation control (dashed line) (Mean±SD, n=3).


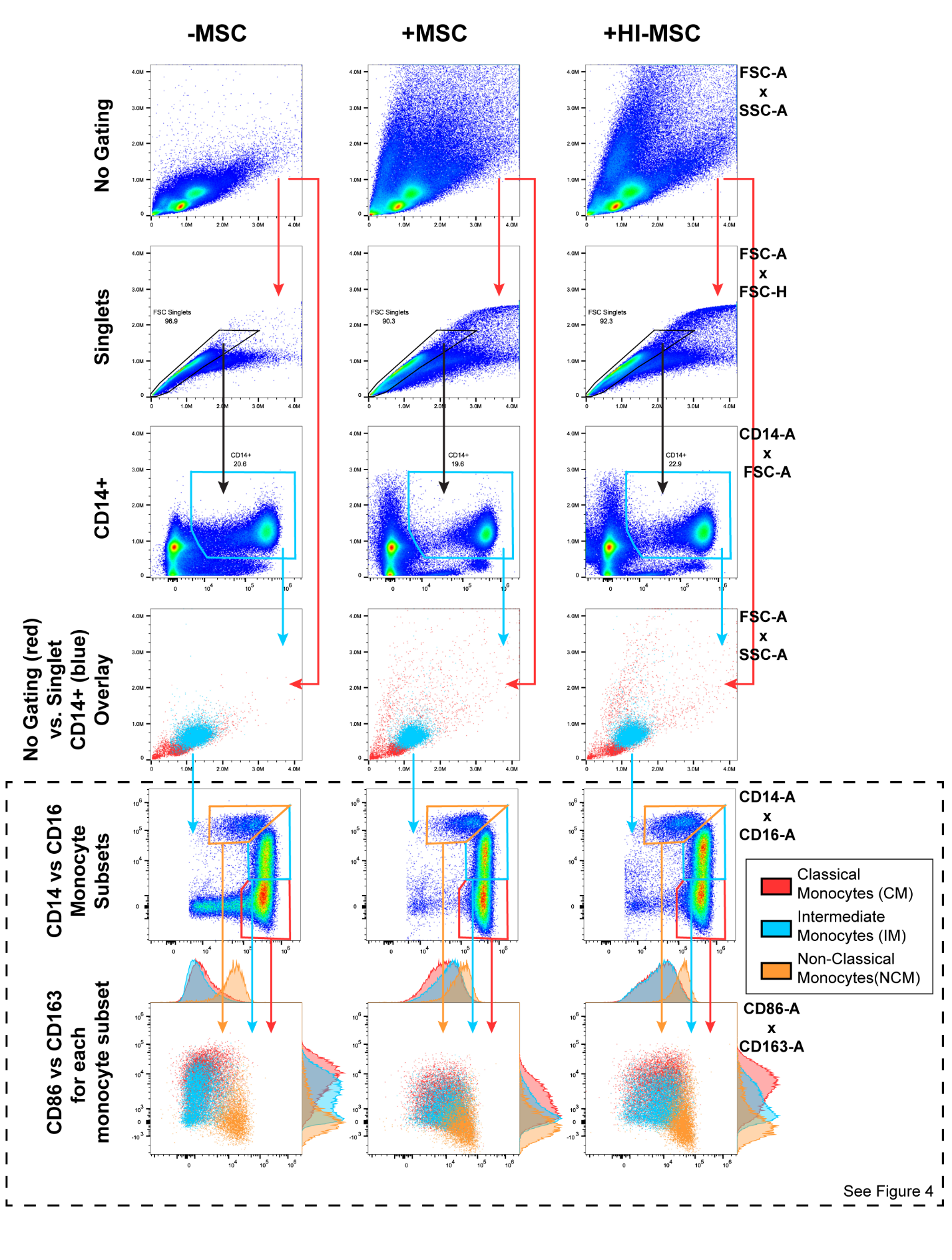


Figure S4: Gating strategy for surface marker analysis of monocytes following MSC efferocytosis. All samples underwent singlet discrimination to remove doublets and clumped cells, followed by monocyte gating by positive CD14-AlexaFluor 488 fluorescence. The fourth row of plots shows singlet CD14+ gated cells (blue) overlayed on non-gated cells (red) to show the effectiveness of gating for monocytes. The singlet CD14+ cells were further gated based on CD14 vs. CD16-PE/Fire 640 to differentiate classical (CM), intermediate (IM, and non-classical monocytes (NCM). Each monocyte subset was assessed for inflammatory status based on CD86 and CD163 MFI. The data from the plots in the dashed-line box are presented in Figure 4 of the original research article.


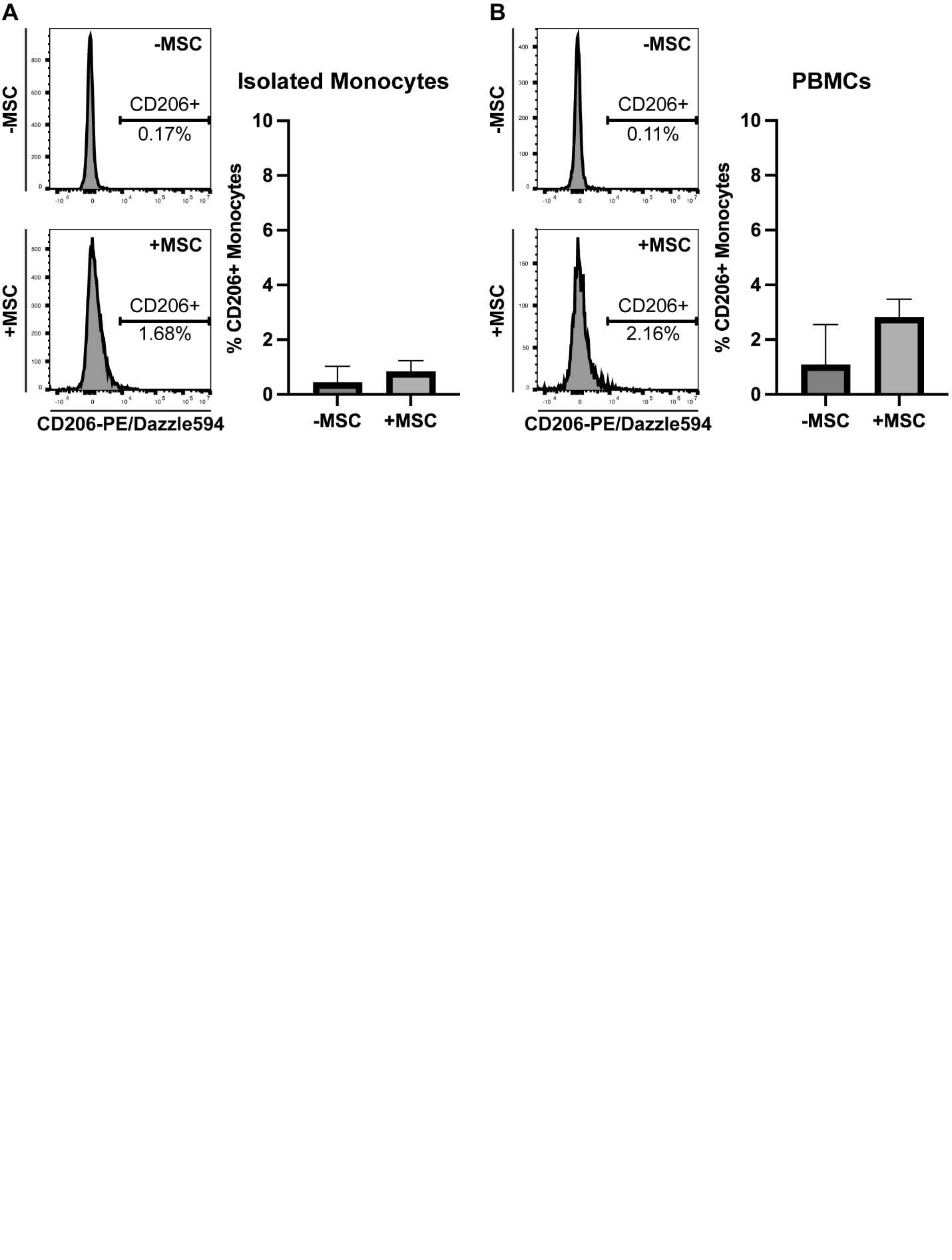


Figure S5: CD206 expression of CD14+ monocytes without and with viable MSC efferocytic-licensing (related to Figure 4). CD14+ monocytes exhibit minimal expression of CD206 without or with MSC efferocytic-licensing in both (A) 1:5 MSC:monocytes and (B) 1:5 MSC:PBMC 24hr cocultures. Monocytes were gated using a fluorescence-minus-one control that contained all surface marker antibodies except CD206. (Mean±SD, n=2).


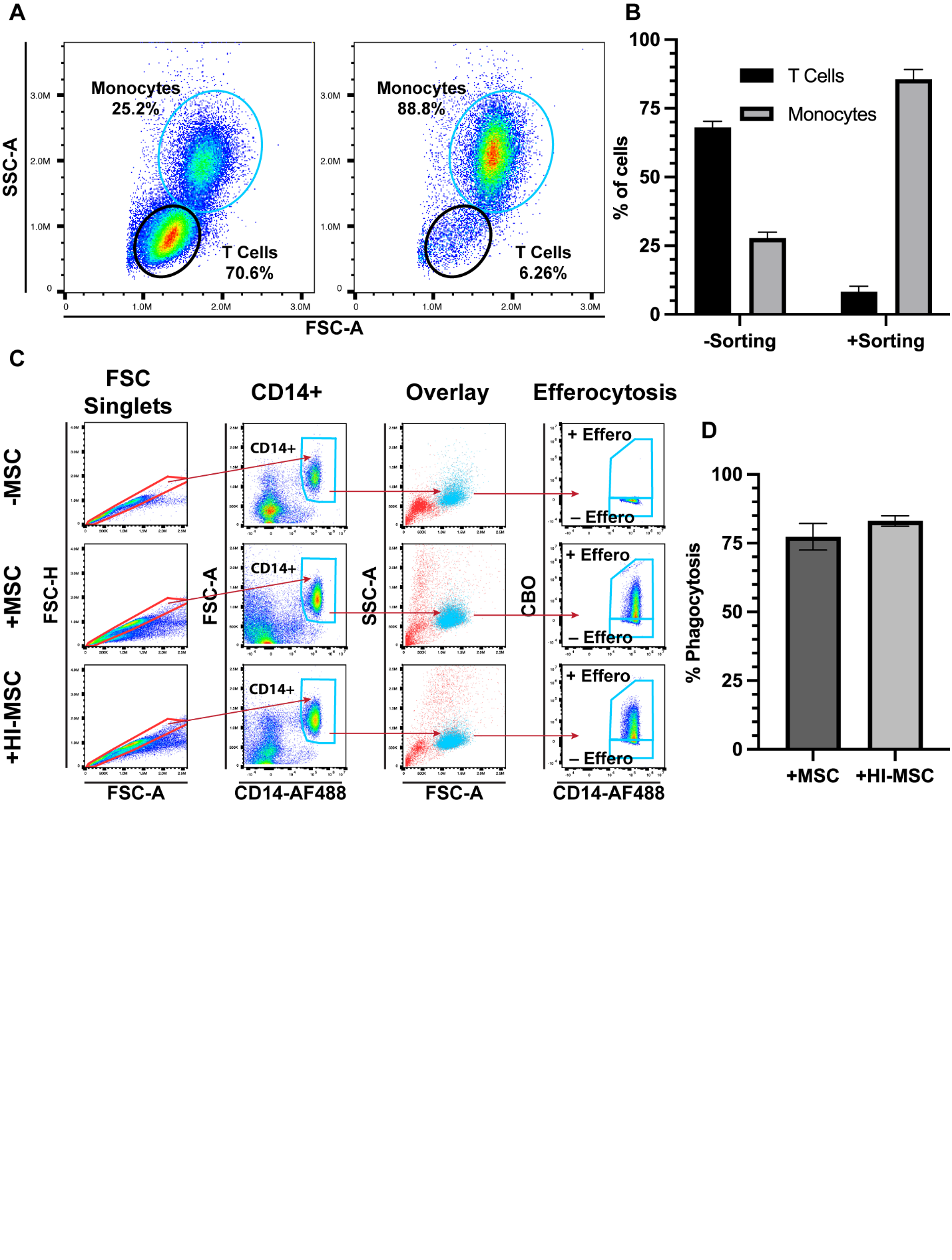


Figure S6: Monocyte isolation is efficient and isolated monocytes phagocytose viable and HI-MSCs in the absence of T-cells(related to Figure 1). (A) Monocyte isolation using a monocyte negative selection magnetic isolation kit results in depletion of non-monocytes, specifically T-cells, based on FSC and SSC. The gates were set using CD14+ monocyte controls. (B) Depletion of non-monocytes from PBMCs results in an isolated monocyte population ~90% (Mean±SD, n=2 independent experiments). (C) Even after T-cell depletion, monocytes still efficiently efferocytose viable- and HI-MSC (1:1 MSC:monocyte) (+Effero). (D) MSC phagocytosis by isolated monocytes results in >75% of monocytes containing MSC material (Mean±SD, n=2 independent experiments).
